# Cholesterol-modified prognostic nutritional index (CPNI) as an effective tool for assessing the nutrition status and predicting survival in patients with breast cancer

**DOI:** 10.1186/s12916-023-03225-7

**Published:** 2023-12-21

**Authors:** Jinyu Shi, Tong Liu, Yizhong Ge, Chenan Liu, Qi Zhang, Hailun Xie, Guotian Ruan, Shiqi Lin, Xin Zheng, Yue Chen, Heyang Zhang, Mengmeng Song, Xiaowei Zhang, Chunlei Hu, Xiangrui Li, Ming Yang, Xiaoyue Liu, Li Deng, Hanping Shi

**Affiliations:** 1 Department of Gastrointestinal Surgery/Department of Clinical Nutrition, Beijing Shijitan Hospital, Capital Medical University, Beijing, 100038 China; 2 Beijing International Science and Technology Cooperation Base for Cancer Metabolism and Nutrition, Beijing, 100038 China; 3 Key Laboratory of Cancer FSMP for State Market Regulation, Beijing, 100038 China; 4 Department of Genetics, Yale School of Medicine, New Haven, CT 06510 USA

**Keywords:** CPNI, Nutrition, Breast cancer, Prognosis

## Abstract

**Background:**

Malnutrition is associated with poor overall survival (OS) in breast cancer patients; however, the most predictive nutritional indicators for the prognosis of patients with breast cancer are not well-established. This study aimed to compare the predictive effects of common nutritional indicators on OS and to refine existing nutritional indicators, thereby identifying a more effective nutritional evaluation indicator for predicting the prognosis in breast cancer patients.

**Methods:**

This prospective study analyzed data from 776 breast cancer patients enrolled in the “Investigation on Nutritional Status and its Clinical Outcome of Common Cancers” (INSCOC) project, which was conducted in 40 hospitals in China. We used the time-dependent receiver operating characteristic curve (ROC), Kaplan–Meier survival curve, and Cox regression analysis to evaluate the predictive effects of several nutritional assessments. These assessments included the patient-generated subjective nutrition assessment (PGSGA), the global leadership initiative on malnutrition (GLIM), the controlling nutritional status (CONUT), the nutritional risk index (NRI), and the prognostic nutritional index (PNI). Utilizing machine learning, these nutritional indicators were screened through single-factor analysis, and relatively important variables were selected to modify the PNI. The modified PNI, termed the cholesterol-modified prognostic nutritional index (CPNI), was evaluated for its predictive effect on the prognosis of patients.

**Results:**

Among the nutritional assessments (including PGSGA, GLIM, CONUT, NRI, and PNI), PNI showed the highest predictive ability for patient prognosis (time-dependent ROC = 0.58). CPNI, which evolved from PNI, emerged as the superior nutritional index for OS in breast cancer patients, with the time-dependent ROC of 0.65. It also acted as an independent risk factor for mortality (*p* < 0.05). Moreover, the risk of malnutrition and mortality was observed to increase gradually among both premenopausal and postmenopausal age women, as well as among women categorized as non-overweight, overweight, and obese.

**Conclusions:**

The CPNI proves to be an effective nutritional assessment tool for predicting the prognosis of patients with breast cancer.

**Supplementary Information:**

The online version contains supplementary material available at 10.1186/s12916-023-03225-7.

## Background

Breast cancer is the most common malignant tumor in women, and its incidence is increasing globally, posing a significant threat to women’s health and life [1]. Malnutrition can lead to decreased immunity, metabolic disorders, and decreased treatment tolerance, subsequently affecting the effectiveness of cancer treatments and patient prognosis [2–7]. Thus, evaluating the nutritional status of patients with breast cancer and implementing appropriate interventions are of great significance for improving their quality of life and prolonging their survival.

Currently, nutritional assessment indicators primarily include patient-generated subjective nutrition assessment (PGSGA), global leadership initiative on malnutrition (GLIM), controlling nutritional status (CONUT), nutritional risk index (NRI), and prognostic nutritional index (PNI) [8–12]. The PGSGA, which primarily relies on patients’ subjective evaluations, includes the assessment of physical function, nutritional status, and metabolic stress, and is known to accurately reflect the patient’s nutritional status. However, its evaluation results may be affected by the subjectivity of the patient’s self-evaluation. The GLIM criteria, widely acknowledged as the global guidelines for nutritional assessment, facilitate the identification of malnutrition in patients; however, they require an evaluation of body weight changes over time, thus lengthening the evaluation process [13]. Moreover, while the CONUT, NRI, and PNI indicators are based on laboratory biochemical indicators and offer simplicity and objectivity, their effectiveness in specific populations remains to be established [14, 15]. Additionally, due to physiological differences, like estrogen levels and body fat content, there is observed variation in the nutritional status between premenopausal and postmenopausal age women. Therefore, the applicability of these indicators for breast cancer patients warrants further investigation.

This large-scale, multicentre, population-based cohort study investigated the relationship between multiple nutritional assessment indicators and mortality in breast cancer patients. We compared the predictive effect of these indicators on patients’ overall survival (OS). Additionally, we refined the existing nutritional indicators to identify the optimal nutritional assessment indicator to predict the prognosis of breast cancer. Ultimately, this study aims to provide more accurate indicators for the nutritional assessment of breast cancer patients and better guidance to clinicians.

## Methods

### Study population

This study was based on the “Investigation on Nutritional Status and its Clinical Outcomes of Common Cancers” (INSCOC) project, which was registered at chictr.org.cn (registration number ChiCTR1800020329). The INSCOC project prospectively collected clinical data of patients with cancer in more than 40 hospitals in China. In this study, we initially screened 2,775 female breast cancer patients who visited the clinic between June 2012 and June 2021. Of these, we excluded 1999 patients due to incomplete clinical or survival data, resulting in 776 patients for the final data analysis. All patients were older than 18 years and had complete clinical data and follow-up information. This study was approved by the institutional review boards of all participating institutions and was conducted according to the guidelines of the Declaration of Helsinki. All enrolled patients provided informed consent for the use of their clinical data, and their personal information was anonymized. A flowchart detailing the screening of the study patients is presented in Additional file 1: Fig. S1.

### Patient characteristics and outcomes

Demographic information, clinical parameters, laboratory tests, and physical measurements of all included patients at baseline were comprehensively collected. This includes age, smoking status, drinking status, comorbidities, family history of cancer, tumor pathology, tumor stage, treatment, biochemical indicators, and anthropometric indicators such as height and weight. All these data were obtained from the electronic medical record system. Patients were classified into premenopausal (< 50 years old) and postmenopausal (≥ 50 years old) categories based on their age at diagnosis. For all patients, the body mass index (BMI) was calculated using the formula: BMI = weight (kg)/height (m)^2^. The patients were then divided into three groups according to the BMI: normal weight (< 24 kg/m^2^), overweight (24.0–28.0 kg/m^2^), and obese (≥ 28 kg/m^2^). All these demographic and clinical pathological data were collected at the initial inclusion in the INSCOC project.

The primary endpoint of this study was OS, defined as the duration from diagnosis until death from any cause. Patient survival information was sourced through regular telephone contacts, outpatient visits, or hospitalizations. The follow-up process continued until either the patient's death or the point at which we could no longer contact the patient.

### Malnutrition assessment

Trained staff assessed and recorded the PGSGA at baseline. In addition, we reassessed the GLIM, CONUT, NRI, and PNI based on data collected during the baseline period (Additional file 1: Table S1). The GLIM diagnostic criteria include etiological criteria (reduced food intake or assimilation, inflammation or disease burden) and phenotypic criteria (weight loss, low BMI, and reduced muscle mass). Patients are diagnosed with malnutrition when they meet at least one etiological and one phenotypic criterion. Since all the cancer patients in our study met at least one etiological criterion, our focus was primarily on the phenotypic criteria [16]. According to the GLIM criteria, weight loss is considered significant if it is greater than 5% within 6 months. A BMI of < 20 and < 22 kg/m^2^ for those aged < 70 and ≥ 70 years, respectively, was deemed low. Muscle loss is indicated by a calf circumference (CC), weight-standardized hand grip strength, or mid-arm muscle circumference (MAMC) < 15 percentile (P15) in women. The P15 values for CC, weight-standardized handgrip strength, and MAMC are 29, 0.2144, and 17.06 cm in women, respectively. The CONUT scores are determined from the albumin, lymphocyte, and total cholesterol levels [17]. Albumin levels > 35, 30–34, 25–29, and < 25 g/L; lymphocyte counts ≥ 1.6, 1.2–1.59, 0.8–1.19, and < 0.8 *10^9/L^; and total cholesterol levels ≥ 180, 140–180, 100–139 mmol/L, and < 100 mmol/L are assigned scored as 0, 2, 4, and 6 points, respectively. The scores of albumin, lymphocyte, and total cholesterol levels are then combined. A total score of ≥ 2 points indicates malnutrition. The NRI and PNI are calculated using the following formulas: NRI = 1.519 × albumin (g/L) + 41.7 × current weight/ideal body weight (IBW) [17]. IBW = [height (m)]^2^ × 22. PNI = albumin (g/L) + 5 × lymphocyte count (× 10^9^) [18].

### Statistical analysis

Continuous variables were expressed as mean ± standard deviation (± SD) or median (interquartile range). Continuous variables with normal distribution were evaluated using Student’s *t*-test, while continuous variables with non-normal distribution were tested using the Mann–Whitney *U* test. Categorical variables were presented as frequencies or percentages, and *χ*^2^ tests or Fisher’s exact tests were applied. Continuous nutritional indicators were dichotomized based on optimal cut-offs, determined using maximally rank statistics. The time-dependent receiver operating characteristic curve (ROC) was utilized to evaluate the predictive power of the different nutritional assessment indices for OS. A machine learning method was employed to screen variables and to construct new and improved indicators. Restricted cubic spline (RCS) plots were used to explore the associations between the modified malnutrition indicators and OS. Kaplan–Meier curves and log-rank tests were used to compare survival between the groups. Univariate and multivariate Cox regression analyses were conducted to analyze the independent prognostic value of nutritional indicators for OS in breast cancers. In the Cox regression analyses, model a represented the univariate regression model; model b included tumor stage and BMI; and model c incorporated tumor stage, BMI, diabetes, hypertension, coronary heart disease, smoking, alcohol consumption, surgery, chemotherapy, and radiation. Statistical significance was established as a two-sided *P*-value < 0.05. All statistical analyses were performed using R version 4.2.1.

## Results

### Patient baseline characteristics

Complete data from 776 breast cancer patients were analyzed in the final analysis. Their median age was 52 (45–61) years, with 317 (40.9%) classified as premenopausal and 459 (59.1%) postmenopausal. A significant proportion of the patients were overweight or obese: 180 (23.2%) were classified as overweight, and 127 (16.4%) as obese. Patients were also categorized by disease stage: 147 (18.9%) were in stage I, 248 (32.0%) in stage II, 132 (17.0%) in stage III, and 249 (32.1%) in stage IV. In addition, we further compared premenopausal and postmenopausal baseline data. Postmenopausal women had a significantly higher BMI than premenopausal women. The baseline patient characteristics are detailed in Table 1.

**Table 1:** The baseline characteristics of the study population

Characteristic	Overall (*n* = 776)	Premenopausal (*n* = 317)	Postmenopausal (*n* = 459)	*p*
Age, years, median (IQR)	52.00 (45.00, 61.00)	44.00 (38.00, 47.00)	59.00 (54.00, 64.00)	< 0.001
Smoking, yes, *n* (%)	47 (6.1)	11 (3.5)	36 (7.8)	0.018
Drinking, yes, *n* (%)	15 (1.9)	9 (2.8)	6 (1.3)	0.208
Diabetes, yes, *n* (%)	66 (8.5)	5 (1.6)	61 (13.3)	< 0.001
Hypertension, yes, *n* (%)	113 (14.6)	9 (2.8)	104 (22.7)	< 0.001
Coronary heart disease, yes, *n* (%)	28 (3.6)	1 (0.3)	27 (5.9)	< 0.001
Family history of tumor, yes, *n* (%)	134 (17.3)	57 (18.0)	77 (16.8)	
Tumor stage, *n* (%)				0.835
I	147 (18.9)	63 (19.9)	84 (18.3)	
II	248 (32.0)	99 (31.2)	149 (32.5)	
III	132 (17.0)	57 (18.0)	75 (16.3)	
IV	249 (32.1)	98 (30.9)	151 (32.9)	
Surgery, *n* (%)	94 (12.1)	38 (12.0)	56 (12.2)	1
Chemotherapy, *n* (%)	508 (65.5)	216 (68.1)	292 (63.6)	0.22
Radiotherapy, *n* (%)	43 (5.5)	18 (5.7)	25 (5.4)	1
Hemoglobin, g/L, median (IQR)	123.00 (112.00, 133.00)	121.00 (110.00, 131.00)	125.00 (113.00, 135.00)	0.001
WBC, 10^9^/L, median (IQR)	5.40 (4.37, 6.73)	5.32 (4.30, 6.60)	5.44 (4.42, 6.86)	0.268
Neutrophil, 109/L, median (IQR)	3.19 (2.40, 4.27)	3.16 (2.33, 4.20)	3.20 (2.44, 4.33)	0.497
Lymphocyte, 109/L, median (IQR)	1.56 (1.22, 1.99)	1.52 (1.19, 1.95)	1.58 (1.25, 2.01)	0.139
Platelets, 109/L, median (IQR)	234.00 (189.00, 290.00)	246.73 (198.00, 297.00)	226.00 (182.00, 278.00)	0.002
Cholesterol, mmol/L, median (IQR)	4.75 (4.18, 5.47)	4.52 (3.93, 5.19)	4.93 (4.34, 5.66)	< 0.001
HDL, mmol/L, median (IQR)	1.27 (1.08, 1.51)	1.29 (1.13, 1.57)	1.25 (1.06, 1.49)	0.038
LDL, mmol/L, median (IQR)	2.85 (2.33, 3.44)	2.67 (2.24, 3.26)	2.99 (2.42, 3.53)	< 0.001
Triglyceride, mmol/L, median (IQR)	1.57 (1.11, 2.14)	1.41 (0.99, 1.99)	1.68 (1.19, 2.28)	< 0.001
Blood glucose, mmol/L, median (IQR)	5.26 (4.82, 5.82)	5.05 (4.67, 5.50)	5.48 (4.91, 6.11)	< 0.001
Total protein, g/L, median (IQR)	69.50 (65.00, 73.40)	69.40 (64.80, 73.60)	69.50 (65.20, 73.30)	0.551
Albumin, g/L, median (IQR)	40.95 (37.60, 43.90)	41.20 (37.80, 44.00)	40.70 (37.55, 43.80)	0.251
Tbil, μmol/L, median (IQR)	9.30 (6.60, 12.20)	8.60 (6.30, 11.30)	9.50 (7.05, 12.60)	0.004
Dbil, μmol/L, median (IQR)	2.70 (2.10, 3.60)	2.60 (2.00, 3.70)	2.80 (2.10, 3.50)	0.621
AST, U/L, median (IQR)	22.00 (18.00, 29.00)	21.60 (17.40, 28.00)	22.60 (18.85, 29.80)	0.033
ALT, U/L, median (IQR)	19.00 (13.00, 29.42)	18.90 (12.10, 30.00)	19.00 (13.80, 29.10)	0.146
Creatinine, μmol/L, median (IQR)	57.00 (51.00, 63.00)	56.10 (51.00, 62.00)	57.40 (50.95, 63.65)	0.342
BUN, mmol/L, median (IQR)	4.70 (3.87, 5.78)	4.36 (3.67, 5.24)	4.95 (4.01, 6.06)	< 0.001
Height, cm, median (IQR)	158.0 (155.0, 162.0)	158.0 (155.0, 162.0)	158.0 (155.0, 161.0)	0.116
Weight, kg, median (IQR)	58.65 (54.00, 65.23)	57.00 (52.50, 64.00)	61.00 (55.00, 67.00)	< 0.001
BMI, kg/m^2^, median (IQR)	23.83 (21.60, 26.17)	22.75 (20.70, 25.00)	24.41 (22.07, 26.71)	< 0.001
BMI4group (%)				< 0.001
Underweight	37 (4.8)	20 (6.3)	17 (3.7)	
Normal weight	366 (47.2)	180 (56.8)	186 (40.5)	
Overweight	270 (34.8)	90 (28.4)	180 (39.2)	
Obesity	103 (13.3)	27 (8.5)	76 (16.6)	
MAC, cm, median (IQR)	27.45 (25.00, 29.50)	27.00 (25.00, 29.00)	27.80 (25.60, 30.00)	0.005
TSF, cm, median (IQR)	22.00 (16.00, 28.00)	22.00 (16.00, 28.00)	22.00 (16.00, 26.00)	0.65
MAMC, cm, median (IQR)	20.73 (18.60, 22.60)	20.48 (17.71, 22.54)	20.84 (19.09, 22.68)	0.006
CC, cm, median (IQR)	34.00 (32.00, 36.50)	34.00 (31.50, 36.50)	34.00 (32.00, 36.50)	0.872
Grip, Kg, median (IQR)	20.60 (16.48, 24.70)	22.00 (18.00, 26.00)	19.70 (15.45, 23.70)	< 0.001
Grip strength, median (IQR)	0.34 (0.27, 0.42)	0.38 (0.31, 0.46)	0.32 (0.25, 0.40)	< 0.001
PGSGA, *n* (%)				1
No malnutrition	495 (63.8)	202 (63.7)	293 (63.8)	
Malnutrition	281 (36.2)	115 (36.3)	166 (36.2)	
GLIM, *n* (%)				< 0.001
No malnutrition	611 (78.7)	226 (71.3)	385 (83.9)	
Malnutrition	165 (21.3)	91 (28.7)	74 (16.1)	
CONUT, *n* (%)				0.023
No malnutrition	409 (52.7)	151 (47.6)	258 (56.2)	
Malnutrition	367 (47.3)	166 (52.4)	201 (43.8)	
NRI, *n* (%)				0.653
No malnutrition	665 (85.7)	269 (84.9)	396 (86.3)	
Malnutrition	111 (14.3)	48 (15.1)	63 (13.7)	
PNI, *n* (%)				0.315
No malnutrition	696 (89.7)	289 (91.2)	407 (88.7)	
Malnutrition	80 (10.3)	28 (8.8)	52 (11.3)	
CPNI, *n* (%)				0.004
No malnutrition	436 (56.2)	198 (62.5)	238 (51.9)	
Malnutrition	340 (43.8)	119 (37.5)	221 (48.1)	

*WBC*, white blood cells; *HDL*, high-density lipoprotein cholesterol; *LDL*, low-density lipoprotein cholesterol; *Tbil*, total bilirubin; *Dbil*, direct bilirubin; *AST*, aspartate aminotransferase; *ALT*, glutamate aminotransferase; *BUN*, blood urea nitrogen; *BMI*, body mass index; *MAC*, mid-arm circumference; *TSF*, triceps skinfold; *MAMC*, midarm muscle circumference; *CC*, calf-circumference; *PGSGA*, the patient-generated subjective nutrition assessment; *GLIM*, the global leadership initiative on malnutrition; *CONUT*, the controlling nutritional status; *NRI*, the nutritional risk index, *PNI*, the prognostic nutritional index; *CPNI*, the cholesterol modified prognostic nutritional index

### Prevalence of malnutrition

The RCS based on NRI, PNI indicators in relation to the mortality of breast cancer patients are depicted in Additional file 1: Fig. S2. The optimal cut-off values for NRI and PNI indicators have been identified as 97.5 and 42 points, respectively (Additional file 1: Fig. S3). The percentage of breast cancer patients diagnosed with malnutrition varied, ranging from 10.3% based on the PNI criteria to 47.3% using the PGSGA. Analysis using PGSGA, GLIM, CONUT, NRI, and PNI indicators revealed that 281 (36.2%), 165 (21.3%), 367 (47.3%), 111 (14.3%), and 80 (10.3%) patients, respectively, were diagnosed with malnutrition (Table 1). Among them, only 5 cases were concurrently identified as malnourished based on the evaluation of all 5 indicators (Additional file 1: Fig. S4). Additionally, the prevalence of malnutrition diagnosed using each nutritional index was determined for patients in the premenopausal and postmenopausal groups (Additional file 1: Fig. S5). As depicted in Additional file 1: Fig. S5, under the diagnostic criteria of GLIM, CONUT, and NRI, postmenopausal women exhibited a lower malnutrition prevalence than premenopausal women. Conversely, under the PNI criteria, postmenopausal women showed a higher malnutrition prevalence compared to their premenopausal counterparts. However, the PGSGA diagnostic criteria revealed no significant difference between the two groups. The relationship between BMI and malnutrition varied depending on the diagnostic criteria. For instance: Using the PGSGA criteria, malnutrition prevalence increased with rising BMI. However, under the GLIM and NRI criteria, the prevalence of malnutrition decreased with an increase in BMI. For CONUT and PNI, the highest malnutrition prevalence was observed in non-overweight patients.

### The prognostic ability comparison of nutrition indicators

Kaplan–Meier curves were utilized to explore the association between malnutrition diagnosed using different nutritional indicators and OS (Additional file 1: Figs. S6–S10). Across various groups, including overall breast cancer patients, pre- and post-menopausal patients, non-overweight, overweight, obese patients, and patients in stages I-II, III, and IV, no significant differences in survival curves were observed between patients diagnosed with malnutrition and those without, according to PGSGA and CONUT indicators. For patients in stage I–II, those diagnosed with malnutrition using the GLIM index exhibited lower survival than those without malnutrition. In the overall breast cancer cohort, pre- and post-menopausal groups, non-overweight and overweight categories, and stage IV, patients diagnosed with malnutrition via the NRI index had lower survival rates than those without malnutrition. However, among obese patients, and those in stages I–II and III, no significant difference in survival rates was noted. Similarly, for the overall breast cancer group, pre- and post-menopausal groups, non-overweight and overweight categories, and stage III, patients diagnosed with malnutrition using the PNI index had lower survival rates than those without malnutrition. Yet, for obese patients and those in stages I–II and IV, no significant difference in survival rates was observed.

**Fig. 1:**
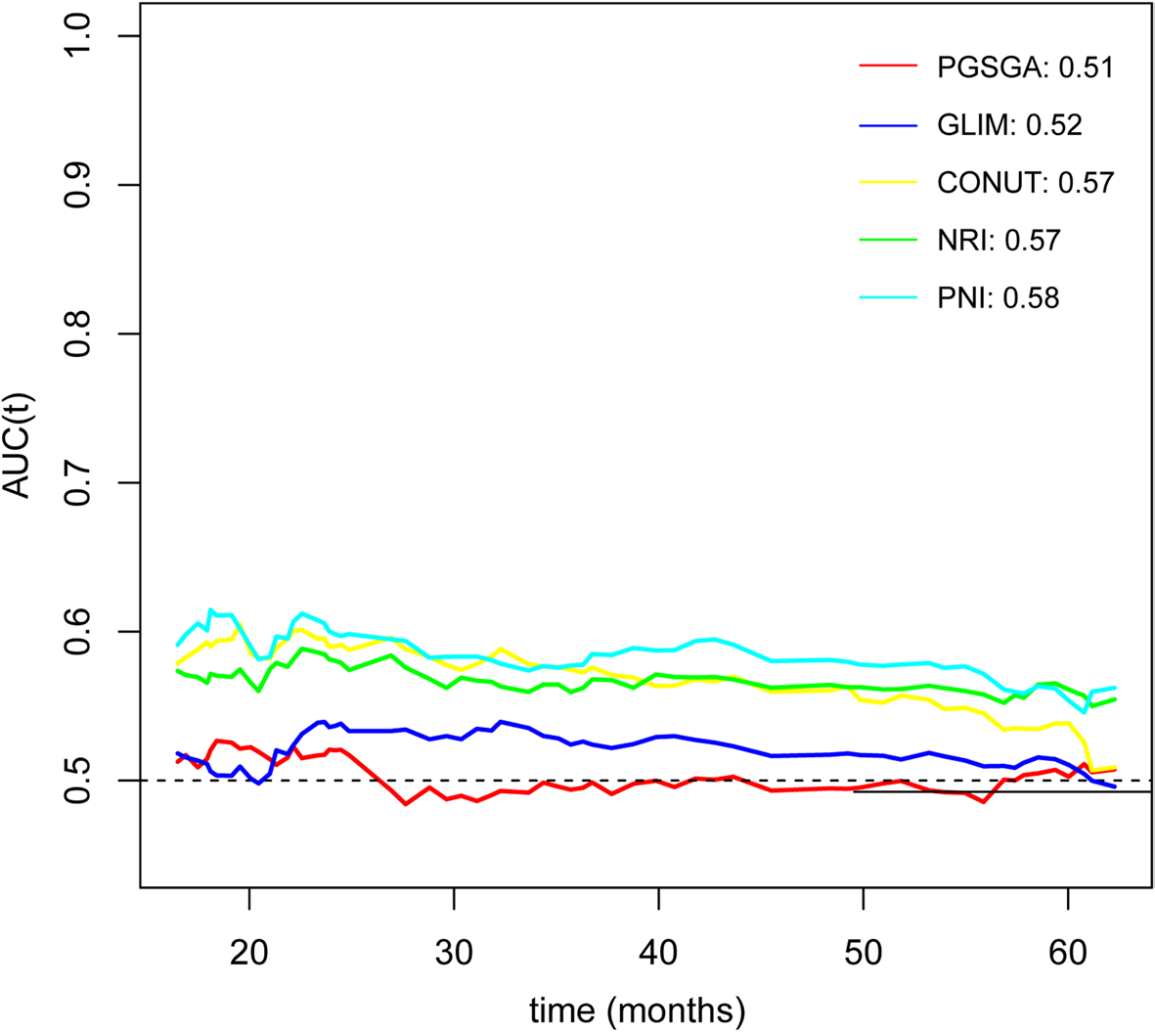
The time-dependent ROC of nutrition-relative indicators for predicting overall survival in patients with breast cancer

In evaluating the prognostic value of PGSGA, GLIM, CONUT, NRI, and PNI in breast cancer patients using the time-dependent ROC, it was determined that PNI had a better predictive value for OS compared to the other nutritional indicators (Fig. 1). Furthermore, upon examining the area under the curve (AUC) of the above 5 indicators at 1, 3, and 5 years, PNI consistently demonstrated better predictive value than the other nutritional indicators (Additional file 1: Table S2).

### Construction of CPNI based on PNI

**Fig. 2:**
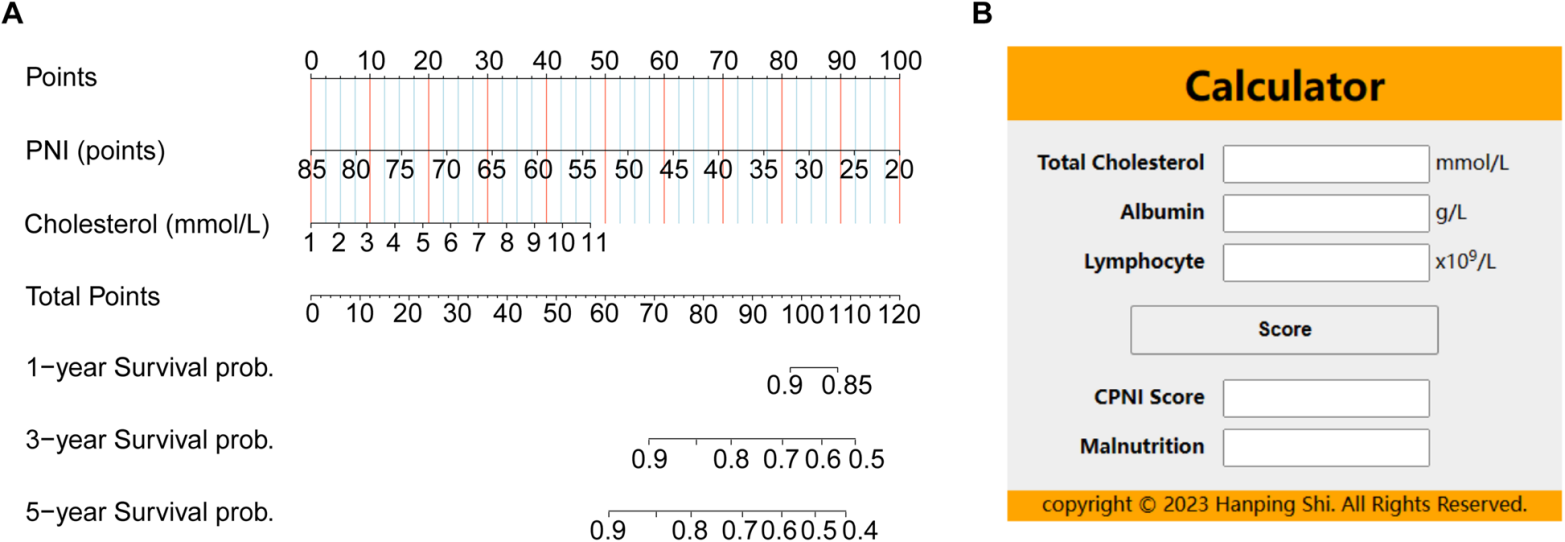
Nomogram and calculator for predicting overall survival of breast cancer patients. Notes: **A** Nomogram. **B** CPNI calculator

Utilizing the PNI as a foundation, we developed an enhanced nutritional index by integrating components from other nutritional indicators. We extracted individual metrics from PGSGA, GLIM, CONUT, and NRI, and then assessed their variable importance using machine learning methods (random forest) (Additional file 1: Fig. S11). Notably, total cholesterol was identified as the most significant variable in this ranking. Recognizing its importance, we incorporated total cholesterol into the PNI, thus creating this enhanced nutritional indicator. To provide a clear and practical tool for clinicians and researchers, we subsequently constructed a prognostic nomogram. This visual representation of our predictive model, displayed in Fig. 2A, assigns a specific score on the point line for each component or risk factor included in the nutritional indicators.

Based on the scores derived from the nomogram, the weighted average value of each variable was calculated. Specifically, total cholesterol was allotted 47.5 points, and PNI received 100 points. We formulated the CPNI (cholesterol-modified prognostic nutritional index) as CPNI = (47.5/10) * (cholesterol − 1) + (100/−65) * (PNI − 85), equating to CPNI = 4.8 cholesterol − 1.5 albumin − 7.7 lymphocyte + 126. This comprehensive metric provides a nuanced, clinically relevant insight into a patient's nutritional status, particularly in relation to their breast cancer prognosis. To facilitate its application in clinical settings, we have developed an online calculator based on the nomogram model. By entering the necessary data, users can instantly obtain a CPNI score from the calculator, which also assesses the presence or absence of malnutrition (Fig. 2B, Additional file 2).

**Fig. 3:**
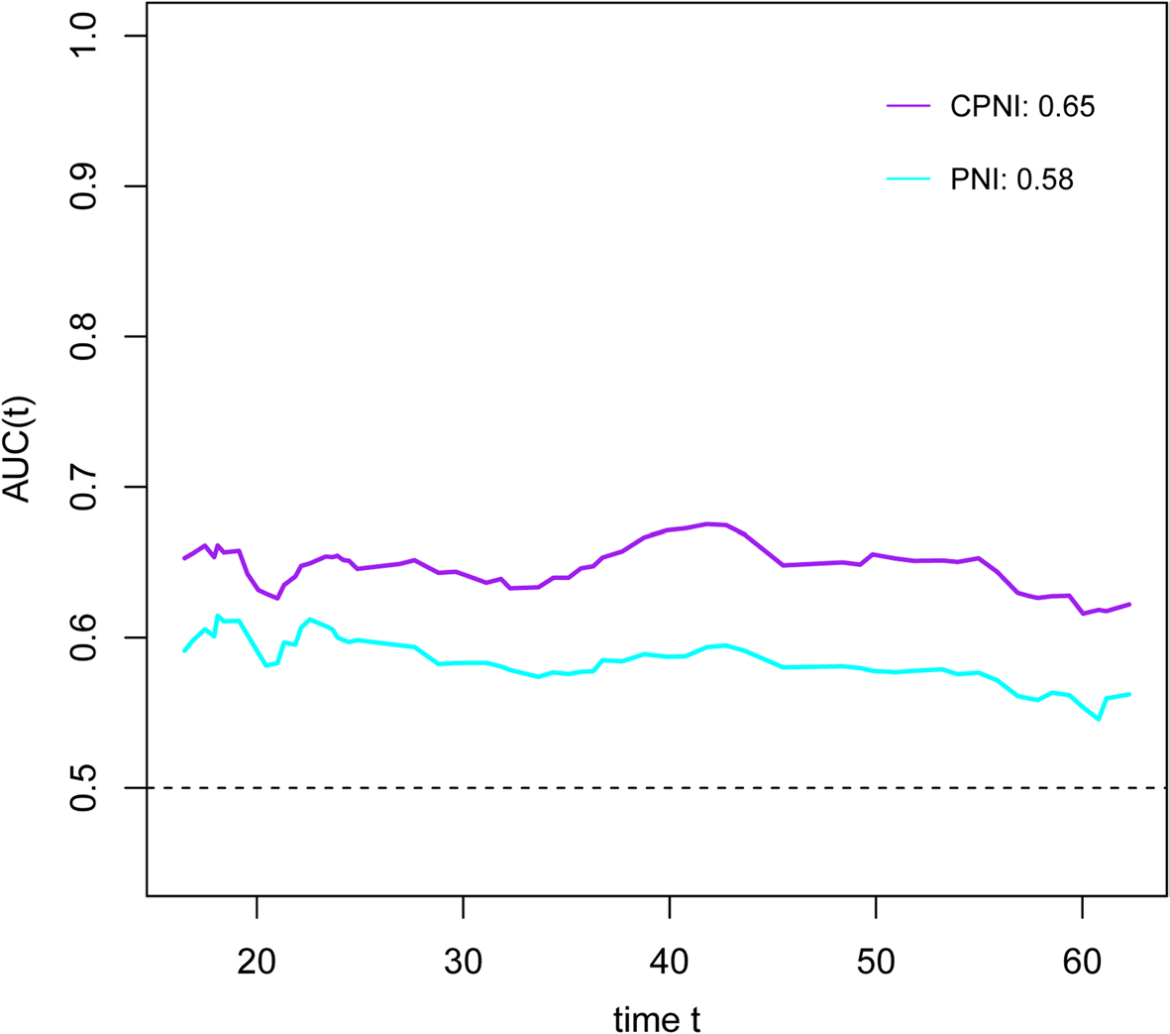
The time-dependent ROC of CPNI and PNI for predicting overall survival in patients with breast cancer

**Fig. 4:**
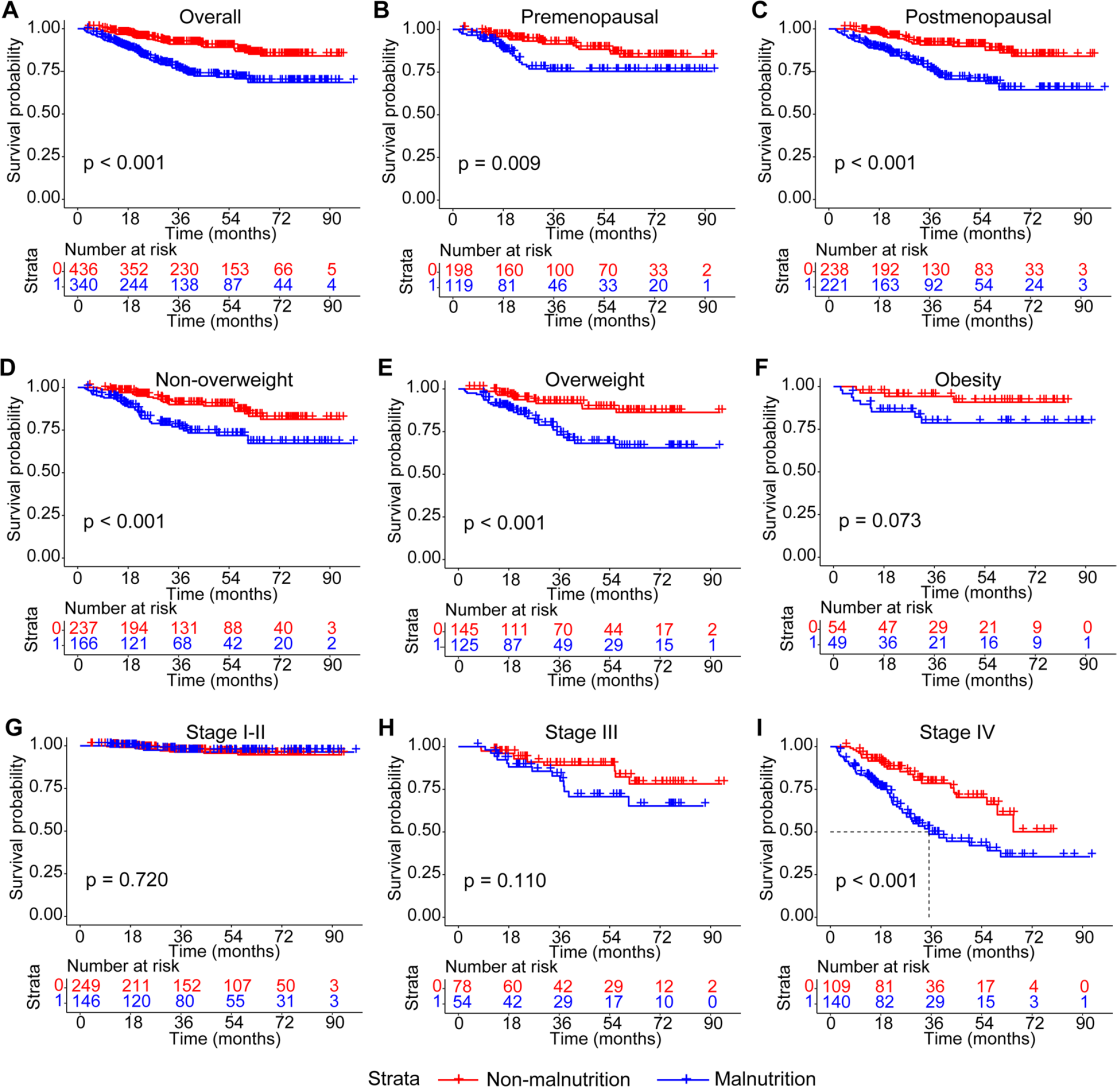
The Kaplan–Meier curves of breast cancer patients with malnutrition and no malnutrition based on the CPNI index. Notes: **A** Total population. **B** Premenopausal patients. **C** Postmenopausal patients. **D** Non-overweight patients. **E** Overweight patients. **F** Obese patients. **G** Stage I–II patients. **H** Stage III patients. **I** Stage IV patients

RCS was employed to examine the correlation between CPNI and mortality. As depicted in Additional file 1: Fig. S12, a positive correlation was observed between CPNI and patient mortality. The optimal cut-off value for CPNI, as depicted in Additional file 1: Fig. S13, was established at 73.72 points. Using this threshold, 340 patients (43.8%) were classified as malnourished. The time-dependent ROC for CPNI was calculated at 0.65, exceeding those of the other nutritional indicators examined (see Fig. 3). Kaplan–Meier survival curves were generated for patients based on their CPNI-diagnosed nutritional status. As shown in Fig. 4, for the overall patient cohort, both pre- and postmenopausal groups, non-overweight and overweight groups, and stage IV patients, those diagnosed with malnutrition via the CPNI index exhibited shorter survival durations compared to their well-nourished counterparts. Among obese and stage II patients, malnourished individuals tended to have reduced survival, though this trend was less pronounced for stage I-II patients.

### Sensitivity analysis and randomized internal validation

After excluding patients who died within 90 days, the time-dependent ROC analysis further indicated that the CPNI index remained the optimal predictor of breast cancer survival (time-dependent ROC = 0.64) (Additional file 1: Fig. S14). Additionally, we conducted a random internal validation by dividing the total population into the training cohort (543 cases) and the testing cohort (234 cases) at a ratio of 7:3 by using a random number generator. Additional file 1: Fig. S15 shows that, in both the training cohort and the testing cohort, the CPNI index consistently emerged as the most effective indicator of survival for breast cancer patients (time-dependent ROC were 0.63 and 0.68, respectively).

### Relationship between nutritional indicators and mortality

Univariate and multivariate Cox regression analyses revealed no significant correlation between PGSGA, GLIM, and CONUT indicators and mortality for overall breast cancer patients, as well as premenopausal and postmenopausal patients (Table 2). Both NRI and PNI indicators identified malnutrition as an independent risk factor for mortality in overall breast cancer patients and premenopausal patients. For postmenopausal breast cancer patients, while univariate analysis suggested a correlation of NRI and PNI indicators with mortality, this correlation was not observed in model b or model c according to the Cox regression. Further exploring the relationship between the CPNI index and mortality, both univariate and multivariate Cox regression analyses indicated the CPNI index as an independent risk factor for mortality in overall, premenopausal, and postmenopausal breast cancer patients.

**Table 2:** The univariate and multivariate Cox analysis for the associations between 6 nutrition-relative indicators and all-cause mortality in patients with breast cancer

	Model a		Model b		Model c	
	HR (95% CI)	*p*	HR (95% CI)	*p*	HR (95% CI)	*p*
**PGSGA**						
Total (*n* = 776)	0.96 (0.66–1.40)	0.827	0.87 (0.60–1.26)	0.457	0.86 (0.59–1.25)	0.422
Premenopausal (*n* = 317)	1.28 (0.70–2.34)	0.432	1.11 (0.60–2.05)	0.73	1.07 (0.58–1.99)	0.828
Postmenopausal (*n* = 459)	0.81 (0.50–1.32)	0.403	0.77 (0.48–1.26)	0.3	0.79 (0.48–1.30)	0.355
**GLIM**						
Total (*n* = 776)	1.07 (0.70–1.65)	0.742	1.04 (0.62–1.74)	0.886	0.97 (0.57–1.63)	0.905
Premenopausal (*n* = 317)	0.81 (0.41–1.62)	0.558	0.85 (0.37–1.96)	0.711	0.77 (0.32–1.81)	0.546
Postmenopausal (*n* = 459)	1.43 (0.82–2.48)	0.205	1.26 (0.66–2.42)	0.484	1.20 (0.61–2.37)	0.59
**CONUT**						
Total (*n* = 776)	1.40 (0.98–2.01)	0.066	0.99 (0.69–1.43)	0.975	0.94 (0.65–1.36)	0.746
Premenopausal (*n* = 317)	1.45 (0.79–2.68)	0.232	1.16 (0.63–2.14)	0.637	1.23 (0.65–2.32)	0.520
Postmenopausal (*n* = 459)	1.40 (0.90–2.20)	0.139	0.91 (0.58–1.44)	0.692	0.82 (0.52–1.29)	0.387
**NRI**						
Total (*n* = 776)	2.03 (1.32–3.12)	0.001	1.96 (1.18–3.25)	0.009	2.10 (1.26–3.49)	0.004
Premenopausal (*n* = 317)	2.42 (1.22–4.81)	0.012	2.55 (1.13–5.75)	0.025	3.47 (1.47–8.17)	0.004
Postmenopausal (*n* = 459)	1.80 (1.04–3.13)	0.037	1.60 (0.83–3.07)	0.160	1.49 (0.77–2.89)	0.242
**PNI**						
Total (*n* = 776)	2.84 (1.83–4.41)	< 0.001	2.08 (1.33–3.25)	0.001	2.03 (1.29–3.19)	0.002
Premenopausal (*n* = 317)	3.39 (1.62–7.07)	0.001	2.73 (1.28–5.83)	0.010	2.85 (1.31–6.19)	0.008
Postmenopausal (*n* = 459)	2.56 (1.47–4.44)	0.001	1.82 (1.04–3.17)	0.035	1.67 (0.94–2.96)	0.080
**CPNI**						
Total (*n* = 776)	2.64 (1.82–3.84)	< 0.001	2.07 (1.42–3.02)	< 0.001	2.04 (1.39–3.00)	< 0.001
Premenopausal (*n* = 317)	2.19 (1.20–3.98)	0.011	1.93 (1.06–3.52)	0.033	1.86 (1.00–3.44)	0.049
Postmenopausal (*n* = 459)	2.96 (1.81–4.83)	< 0.001	2.17 (1.32–3.56)	0.002	2.15 (1.29–3.59)	0.003

Model a: No adjusted

Model b: Adjusted for TNM stage, BMI

Model c: Adjusted for TNM stage, BMI, diabetes, hypertension, coronary heart disease, smoking, drinking, surgery, chemotherapy

Additional file 1: Fig. S16 indicates that malnutrition, as diagnosed by the CPNI index, is linked to a heightened mortality risk in both pre- and postmenopausal women. Intriguingly, Additional file 1: Fig. S17 shows a progressive increase in mortality risk associated with the CPNI across weight classifications: from non-overweight to overweight, and then obese women.

## Discussion

In this study, we explored the correlations between the nutritional indicators PGSGA, GLIM, CONUT, NRI, and PNI with the prognosis in breast cancers to ascertain their prognostic value. Among these five indices, the time-dependent ROC showed that PNI is the most effective predictor for the prognosis of breast cancer patients. Building upon the PNI, we developed a modified index, CPNI, which incorporates total cholesterol, albumin, and lymphocytes. Kaplan–Meier survival curves and Cox regression analyses suggest that CPNI acts as an independent prognostic factor for breast cancers. Furthermore, time-dependent ROC demonstrated that the predictive capability of CPNI exceeds those of the previously mentioned nutritional indicators.

Analysis using the CPNI index revealed that postmenopausal women experience a higher incidence of malnutrition than premenopausal women, corroborating previous research findings. With aging, women undergo numerous physiological changes, especially between the premenopausal and postmenopausal periods. Factors such as a decreased metabolic rate, hormonal fluctuations, and altered eating habits may impact a woman's nutritional status. These can impede the absorption and utilization of essential nutrients like fats, proteins, and trace elements, thereby contributing to elevated malnutrition rates [19]. Concurrently, as age progresses, tolerance to malnutrition decreases, leading to an escalating risk of malnutrition-related mortality. The relationship between malnutrition and BMI is complex. The CPNI analysis indicated an incremental rise in malnutrition across non-overweight to obese patients. While the risk of malnutrition-related mortality increased for non-overweight and overweight patients, it appeared somewhat mitigated in obese patients as compared to their overweight counterparts.

Using the PNI as a foundation, we developed CPNI by screening individual variables within PGSGA, GLIM, CONUT, and NRI, ultimately formulating a new evaluation formula based on weighted averages. The CPNI method offers advantages due to its non-invasiveness, simplicity, objectivity, and suitability for dynamic monitoring. Early detection through CPNI can accurately identify malnutrition and potential poor prognosis, thereby enabling timely clinical interventions. This could substantially enhance patient quality of life and potentially prolong survival. CPNI encompasses three key nutritional indicators: total cholesterol, albumin, and lymphocytes, each closely linked to one's nutritional status. For instance, malnutrition is often signaled by diminished serum cholesterol and albumin levels, as well as reduced lymphocyte counts. These indicators not only reflect nutritional shifts but also highlight the degree of inflammatory response. Several studies have indicated that elevated cholesterol levels might amplify the risk of certain cancers and are associated with more severe malignancy and unfavorable prognoses in tumor patients [20, 21]. Albumin, an integral nutrient reserve, plays a crucial role in vital biological processes, such as regulating immune functions and balancing bodily fluids [22]. Tumor progression often coincides with hypoproteinaemia, attributed to factors like decreased liver synthesis capabilities, inadequate nutrient intake, and metabolic disturbances in cancer patients [23]. Lymphocytes, both in number and functionality, are considered important markers of the nutritional status of tumors [24]. Malnutrition could adversely affect lymphocyte count and effectiveness, thus impairing immune surveillance against tumors. Additionally, tumor cells might produce factors that suppress lymphocytes, further evading immune monitoring. Therefore, in tumor nutritional evaluations, lymphocyte dynamics warrant significant attention. Enhancing lymphocyte count and function through nutritional intervention might strengthen the body's immune surveillance against tumors, potentially leading to improved prognosis.

This study has several limitations that deserve attention. First, the entire study population comprised Chinese patients. Considering racial differences, the extrapolated results of this study may not fully represent a more diverse global population. Second, the absence of a universally accepted gold standard for the diagnosis of malnutrition presents a challenge in comparing the diagnostic accuracy of different nutritional indicators in breast cancers. Third, although we constructed the CPNI indicator based on PNI, the predictive ability of CPNI requires external validation in future studies. Fourth, due to the limited number of underweight patients, this study combined underweight and normal-weight patients into a single non-overweight category for analysis. This approach might obscure the more nuanced relationship between malnutrition and various BMI categories. Additionally, the molecular subtype of breast cancer is closely related to patient prognosis, but the INSCOC project currently lacks molecular subtype data for breast cancer. Investigations into the NHANES, SEER, and Kailuan databases also did not yield comprehensive datasets with both hematological indicators and molecular subtype data for breast cancer. Consequently, subgroup analyses or prognostic analyses of patients with different molecular types could not be conducted, which we plan to further refine in subsequent data collection of the INSCOC project. Fifth, we stratified patients into premenopausal and postmenopausal groups based on age. While this classification approach serves as a common and convenient method to classify menopausal status in large-scale studies, we acknowledge that it may not fully capture the intricacies of the menopausal transition and the individual hormonal changes experienced by patients. However, the INSCOC project currently lacks the specific timing of patient menopause. We will incorporate this information in subsequent studies. In the future, we will also consider more refined menopausal status assessment methods to improve the accuracy of our findings. Finally, the underlying mechanisms of CPNI and breast cancer prognosis have not been fully elucidated, Future laboratory research is planned to investigate this relationship more deeply.

## Conclusions

In conclusion, this study showed that CPNI can be used as an effective nutritional assessment tool to predict the prognosis in breast cancers. Its application is significantly valuable in guiding clinical decision-making and improving patient survival.

### Supplementary Information

**Additional file 1: Figure S1.** The flow chart. **Figure S2.** The association of NRI, PNI and overall survival in patients with breast cancer. **Figure S3.** Cut-off values of NRI and PNI in patients with breast cancer. **Figure S4.** Venn diagram of the numbers of patients with malnutrition diagnosed using different diagnostic criteria. **Figure S5.** Diagnosis rate of different nutrition-relative diagnostic criteria in different age and different BMI groups. Notes: A-F. different age groups. G-L. different BMI groups. **Figure S6.** The Kaplan-Meier curves of breast cancer patients with malnutrition and no malnutrition based on PGSGA index. Notes: A. total population. B. premenopausal patients. C. postmenopausal patients. D. non-overweight patients. E. overweight patients. F. obese patients. G. stage I-II patients. H. stage III patients. I. stage IV patients. **Figure S7.** The Kaplan-Meier curves of breast cancer patients with malnutrition and no malnutrition based on GLIM index. Notes: A. total population. B. premenopausal patients. C. postmenopausal patients. D. non-overweight patients. E. overweight patients. F. obese patients. G. stage I-II patients. H. stage III patients. I. stage IV patients. **Figure S8.** The Kaplan-Meier curves of breast cancer patients with malnutrition and no malnutrition based on CONUT index. Notes: A. total population. B. premenopausal patients. C. postmenopausal patients. D. non-overweight patients. E. overweight patients. F. obese patients. G. stage I-II patients. H. stage III patients. I. stage IV patients. Figure S9. The Kaplan-Meier curves of breast cancer patients with malnutrition and no malnutrition based on NRI index. Notes: A. total population. B. premenopausal patients. C. postmenopausal patients. D. non-overweight patients. E. overweight patients. F. obese patients. G. stage I-II patients. H. stage III patients. I. stage IV patients. **Figure S10.** The Kaplan-Meier curves of breast cancer patients with malnutrition and no malnutrition based on PNI index. Notes: A. total population. B. premenopausal patients. C. postmenopausal patients. D. non-overweight patients. E. overweight patients. F. obese patients. G. stage I-II patients. H. stage III patients. I. stage IV patients. **Figure S11.** Feature selection using the machine learning (random forest). **Figure S12.** The association of CPNI and overall survival in patients with breast cancer. **Figure S13.** Cut-off values of CPNI in patients with breast cancer. **Figure S14.** The time-dependent ROC of nutrition-relative indicators for diagnosing overall survival by excluding patients with short-term deaths (90-days). **Figure S15.** The time-dependent ROC of nutrition-relative indicators for diagnosing overall survival in patients with breast cancer. Notes: A. train cohort. B. test cohort. **Figure S16.** The sub-group analysis of the associations between nutrition indictors and mortality in patients with pre- and post-menopausal. **Figure S17.** The sub-group analysis of the associations between nutrition indictors and mortality in non-overweight, overweight, and obese patients. **Table S1.** Calculation methods for each nutrition indicator. **Table S2.** The AUC of nutrition indicators for all-cause mortality in patients with breast cancer.

**Additional file 2. **CPNI Calcultator.

## Data Availability

The datasets used and analyzed during the current study are available from the corresponding author on reasonable request.

## Abbreviations

AUC: The area under the curve
BMI: Body mass index
CC: Calf circumference
CONUT: Controlling nutritional status
CPNI: Cholesterol-modified prognostic nutritional index
GLIM: Global leadership initiative on malnutrition
IBW: Ideal body weight
INSCOC: Investigation on Nutritional Status and its Clinical Outcome of Common Cancers
MAMC: Mid-arm muscle circumference
NRI: Nutritional risk index
OS: Overall survival
P15: 15 Percentile
PGSGA: Patient-generated subjective nutrition assessment
PNI: Prognostic nutritional index
RCS: Restricted cubic spline
ROC: Receiver operating characteristic curve
