# Supplementary material for: Cholesterol-modified prognostic nutritional index (CPNI) as an effective tool for assessing the nutrition status and predicting survival in patients with breast cancer
Source: BMC Med. 2023 Dec 21;21:512. doi: 10.1186/s12916-023-03225-7 (PMC10740286; doi:10.1186/s12916-023-03225-7)
Supplement: Supplementary file 1 — Additional file 1: Figure S1. The flow chart. Figure S2. The association of NRI, PNI and overall survival in patients with breast cancer. Figure S3. Cut-off values of NRI and PNI in patients with breast cancer. Figure S4. Venn diagram of the numbers of patients with malnutrition diagnosed using different diagnostic criteria. Figure S5. Diagnosis rate of different nutrition-relative diagnostic criteria in different age and different BMI groups. Notes: A-F. different age groups. G-L. different BMI groups. Figure S6. The Kaplan-Meier curves of breast cancer patients with malnutrition and no malnutrition based on PGSGA index. Notes: A. total population. B. premenopausal patients. C. postmenopausal patients. D. non-overweight patients. E. overweight patients. F. obese patients. G. stage I-II patients. H. stage III patients. I. stage IV patients. Figure S7. The Kaplan-Meier curves of breast cancer patients with malnutrition and no malnutrition based on GLIM index. Notes: A. total population. B. premenopausal patients. C. postmenopausal patients. D. non-overweight patients. E. overweight patients. F. obese patients. G. stage I-II patients. H. stage III patients. I. stage IV patients. Figure S8. The Kaplan-Meier curves of breast cancer patients with malnutrition and no malnutrition based on CONUT index. Notes: A. total population. B. premenopausal patients. C. postmenopausal patients. D. non-overweight patients. E. overweight patients. F. obese patients. G. stage I-II patients. H. stage III patients. I. stage IV patients. Figure S9. The Kaplan-Meier curves of breast cancer patients with malnutrition and no malnutrition based on NRI index. Notes: A. total population. B. premenopausal patients. C. postmenopausal patients. D. non-overweight patients. E. overweight patients. F. obese patients. G. stage I-II patients. H. stage III patients. I. stage IV patients. Figure S10. The Kaplan-Meier curves of breast cancer patients with malnutrition and no malnutrition based on PNI index [file 12916_2023_3225_MOESM1_ESM.docx]

Figure S1. The flow chart.


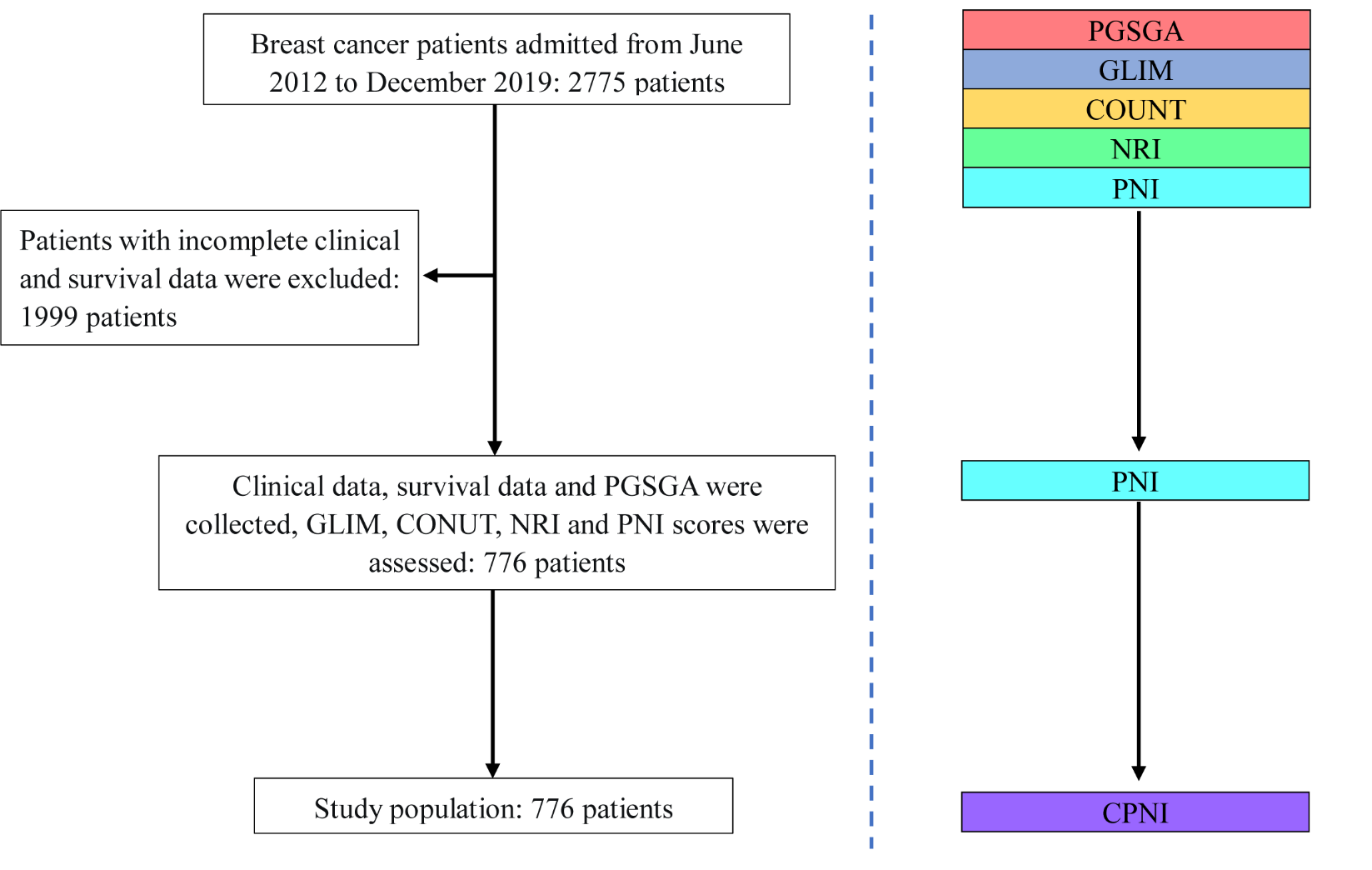


Figure S2. The association of NRI, PNI and overall survival in patients with breast cancer.


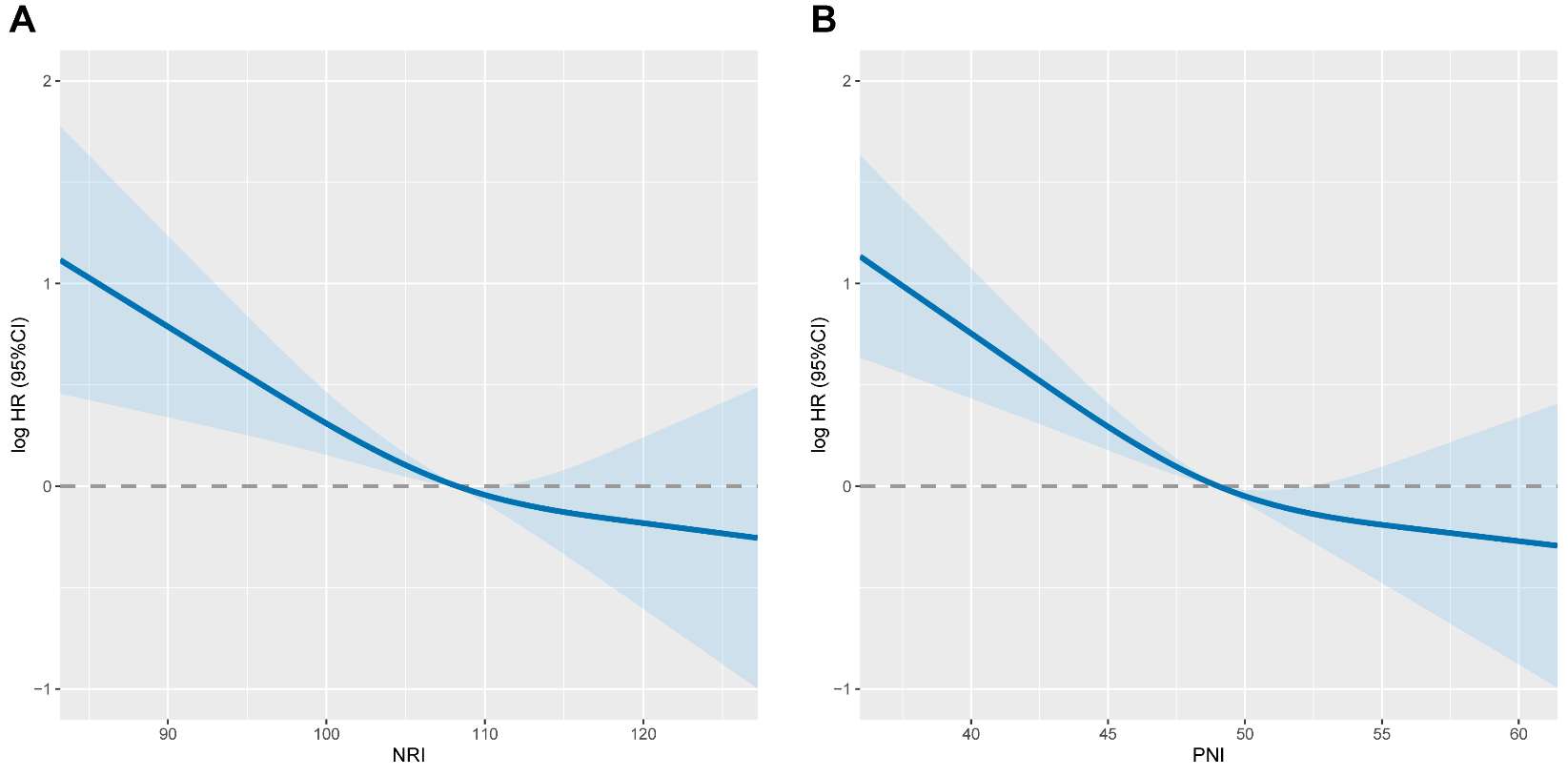


Figure S3. Cut-off values of NRI and PNI in patients with breast cancer.


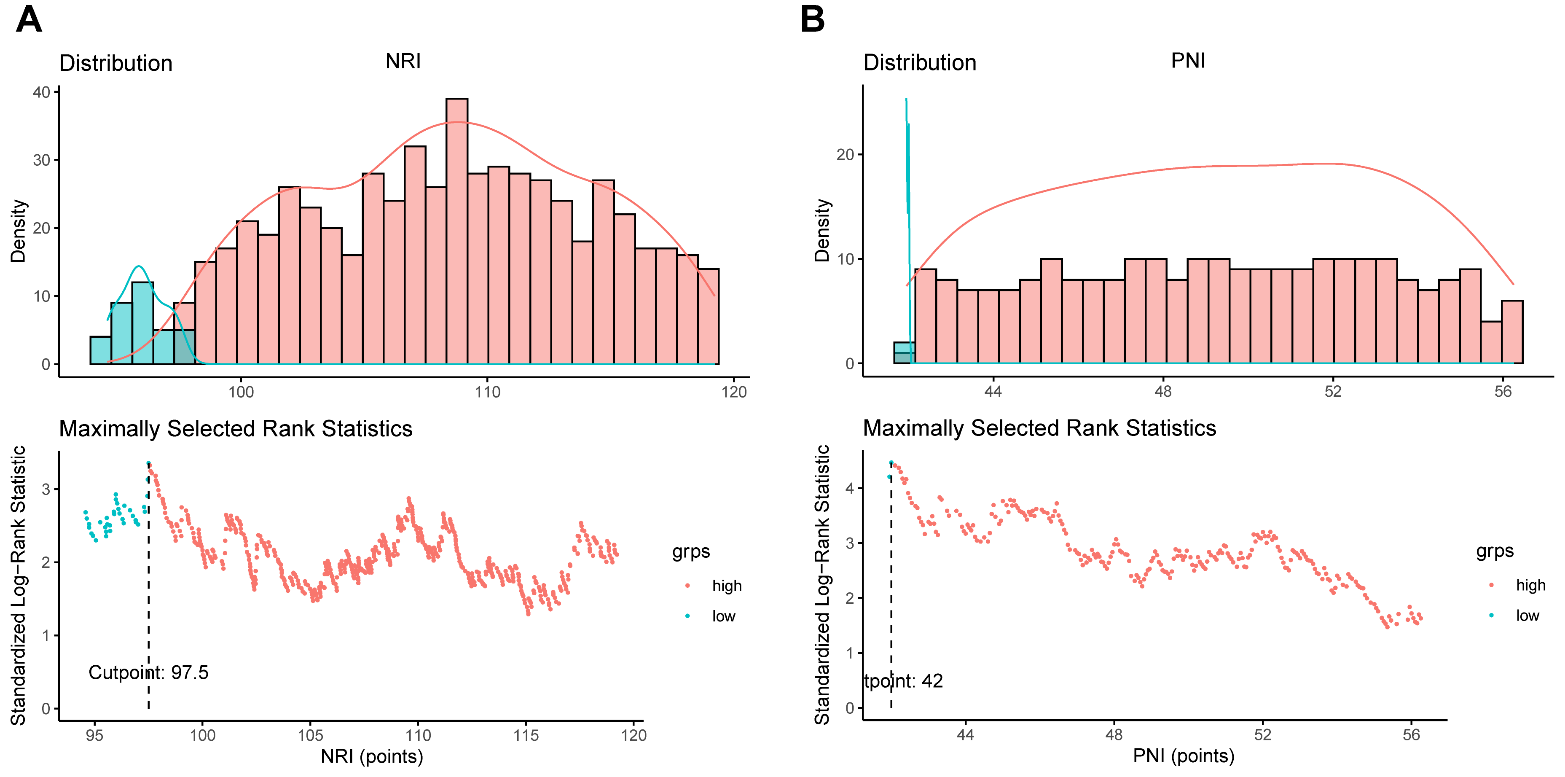


Figure S4. Venn diagram of the numbers of patients with malnutrition diagnosed using different diagnostic criteria.


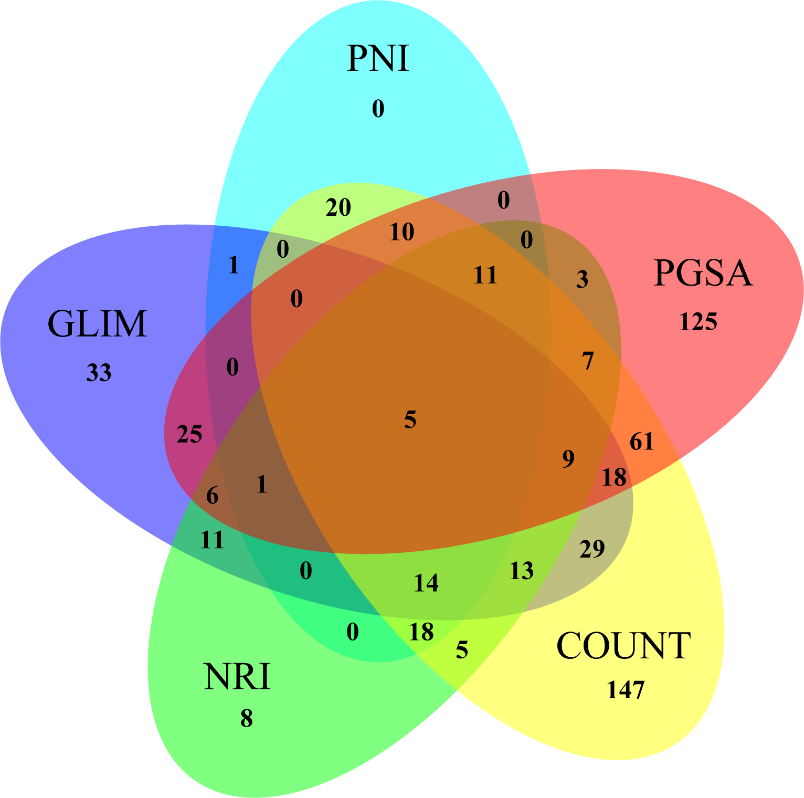


Figure S5. Diagnosis rate of different nutrition-relative diagnostic criteria in different age and different BMI groups.

Notes: A-F. different age groups. G-L. different BMI groups.


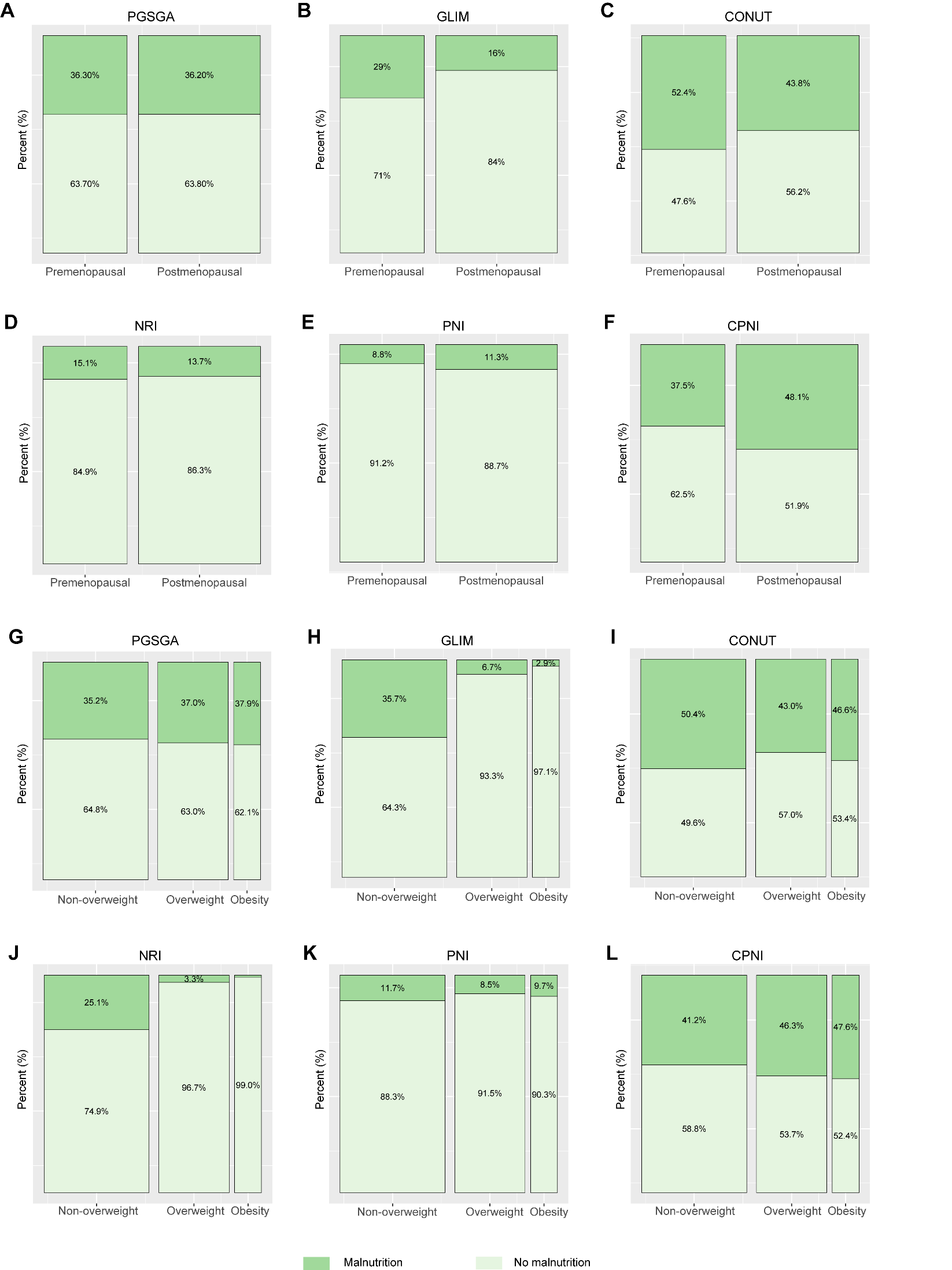


Figure S6. The Kaplan-Meier curves of breast cancer patients with malnutrition and no malnutrition based on PGSGA index.

Notes: A. total population. B. premenopausal patients. C. postmenopausal patients. D. non-overweight patients. E. overweight patients. F. obese patients. G. stage I-II patients. H. stage III patients. I. stage IV patients.


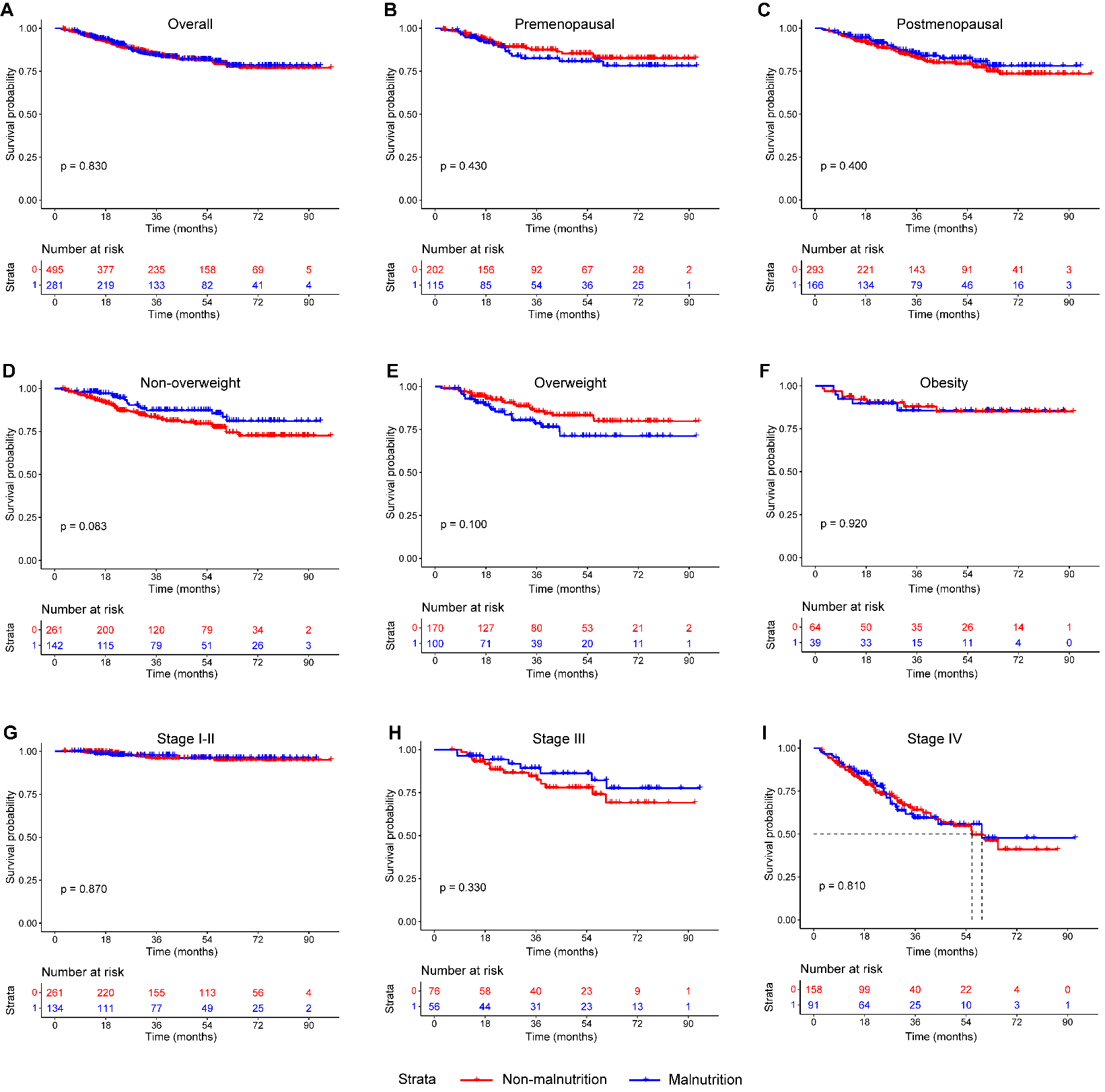


Figure S7. The Kaplan-Meier curves of breast cancer patients with malnutrition and no malnutrition based on GLIM index.

Notes: A. total population. B. premenopausal patients. C. postmenopausal patients. D. non-overweight patients. E. overweight patients. F. obese patients. G. stage I-II patients. H. stage III patients. I. stage IV patients.


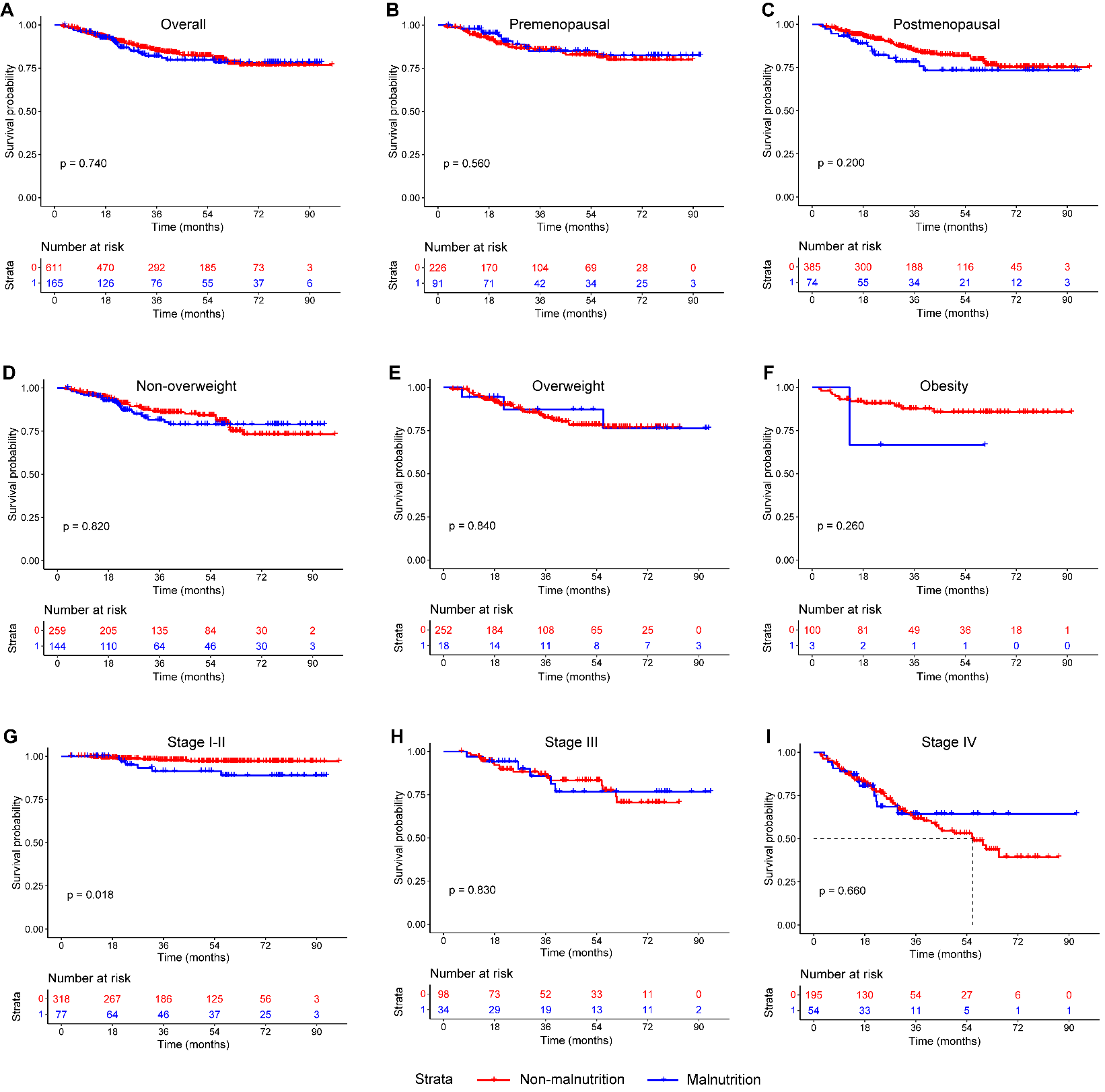


Figure S8. The Kaplan-Meier curves of breast cancer patients with malnutrition and no malnutrition based on CONUT index.

Notes: A. total population. B. premenopausal patients. C. postmenopausal patients. D. non-overweight patients. E. overweight patients. F. obese patients. G. stage I-II patients. H. stage III patients. I. stage IV patients.


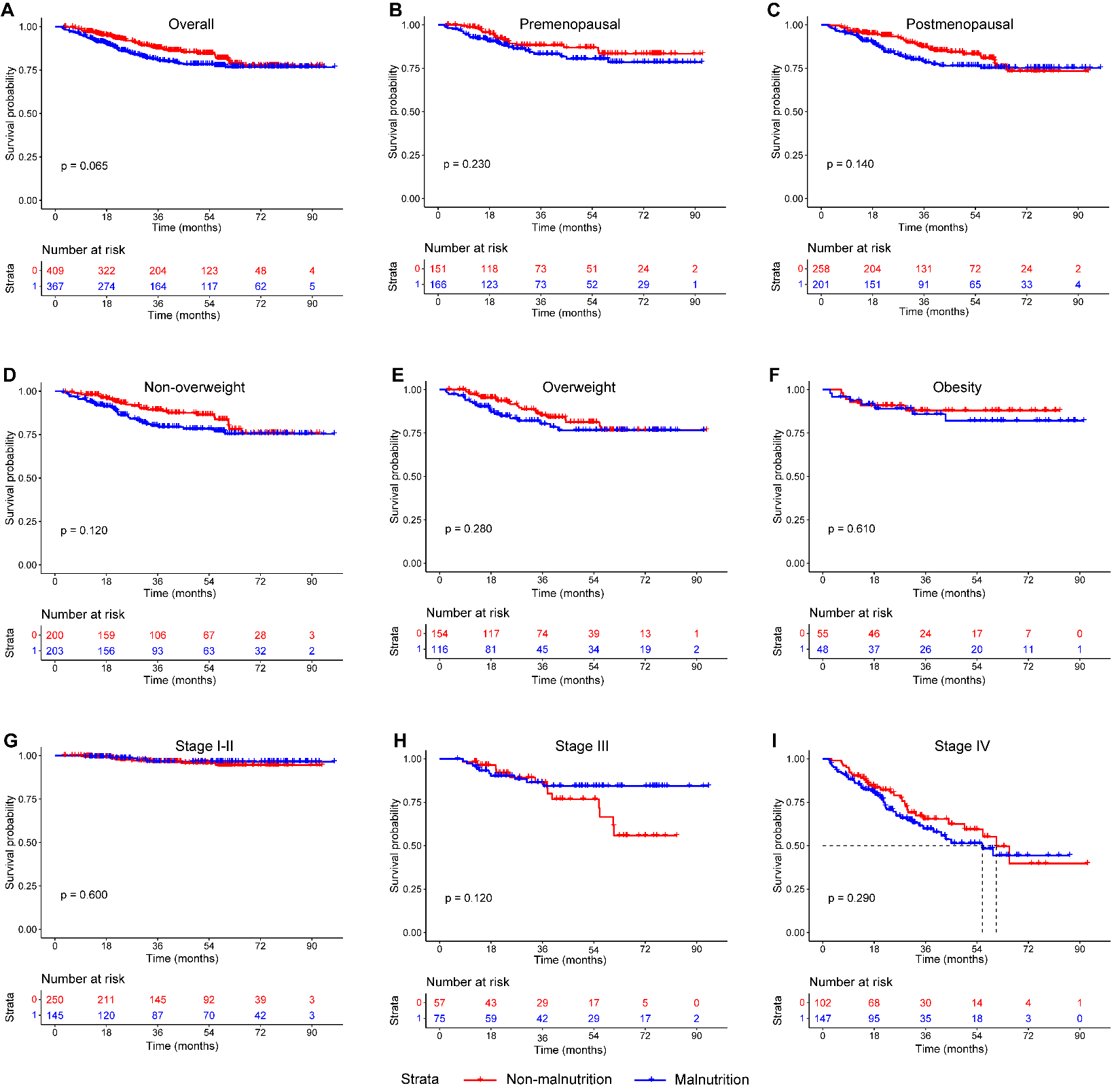


Figure S9. The Kaplan-Meier curves of breast cancer patients with malnutrition and no malnutrition based on NRI index.

Notes: A. total population. B. premenopausal patients. C. postmenopausal patients. D. non-overweight patients. E. overweight patients. F. obese patients. G. stage I-II patients. H. stage III patients. I. stage IV patients.


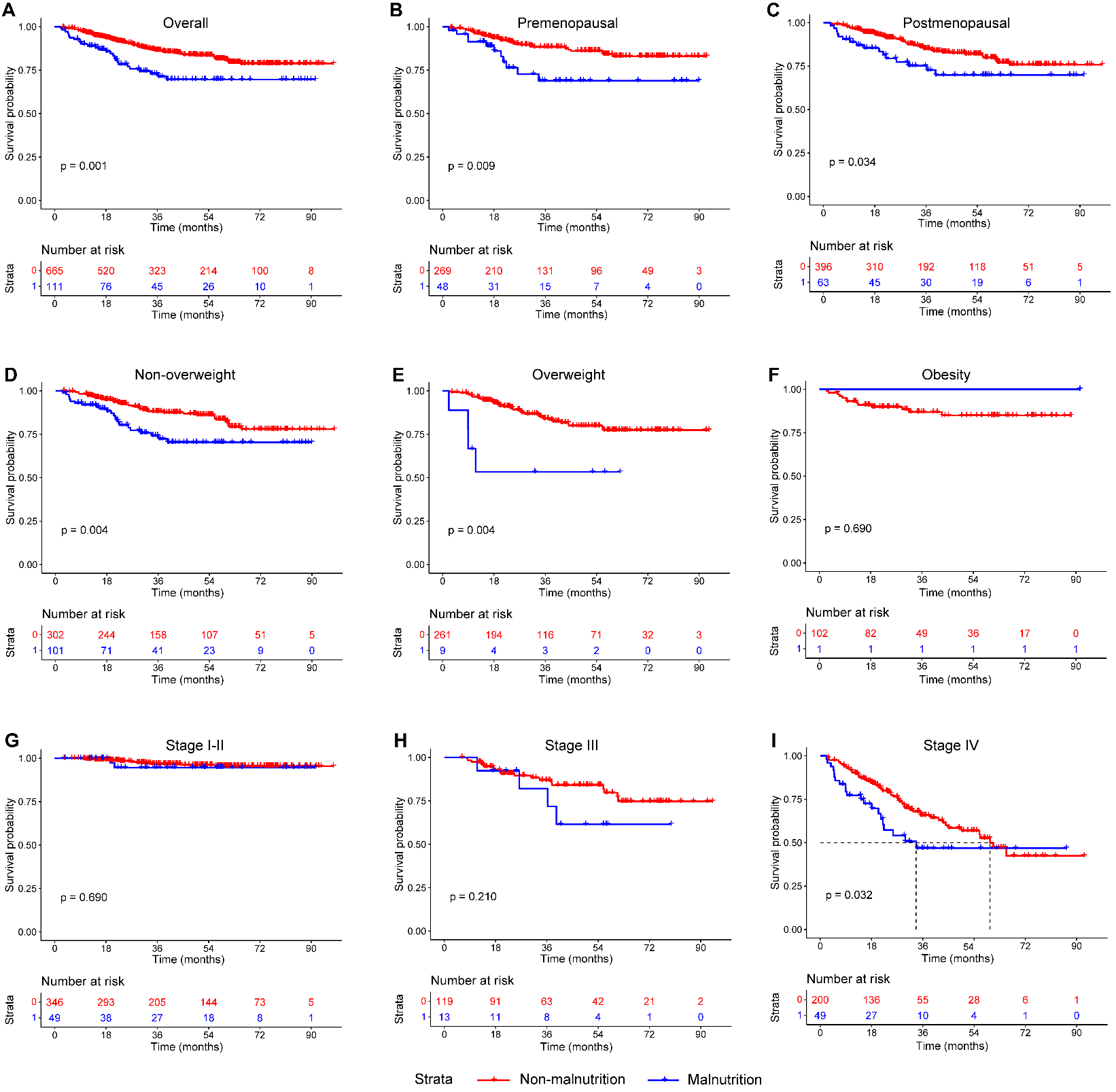


Figure S10. The Kaplan-Meier curves of breast cancer patients with malnutrition and no malnutrition based on PNI index.

Notes: A. total population. B. premenopausal patients. C. postmenopausal patients. D. non-overweight patients. E. overweight patients. F. obese patients. G. stage I-II patients. H. stage III patients. I. stage IV patients.


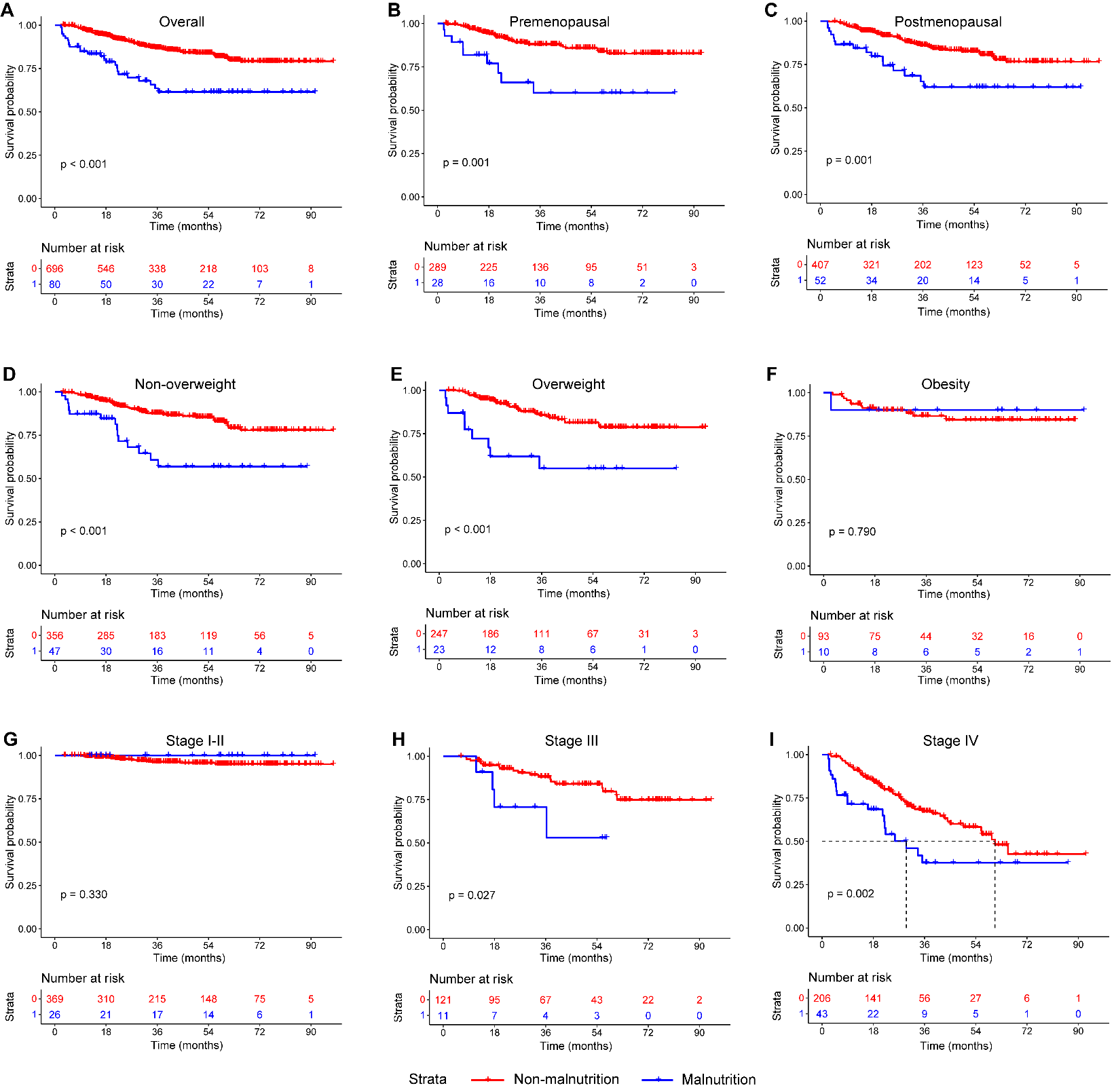


Figure S11. Feature selection using the machine learning (random forest).


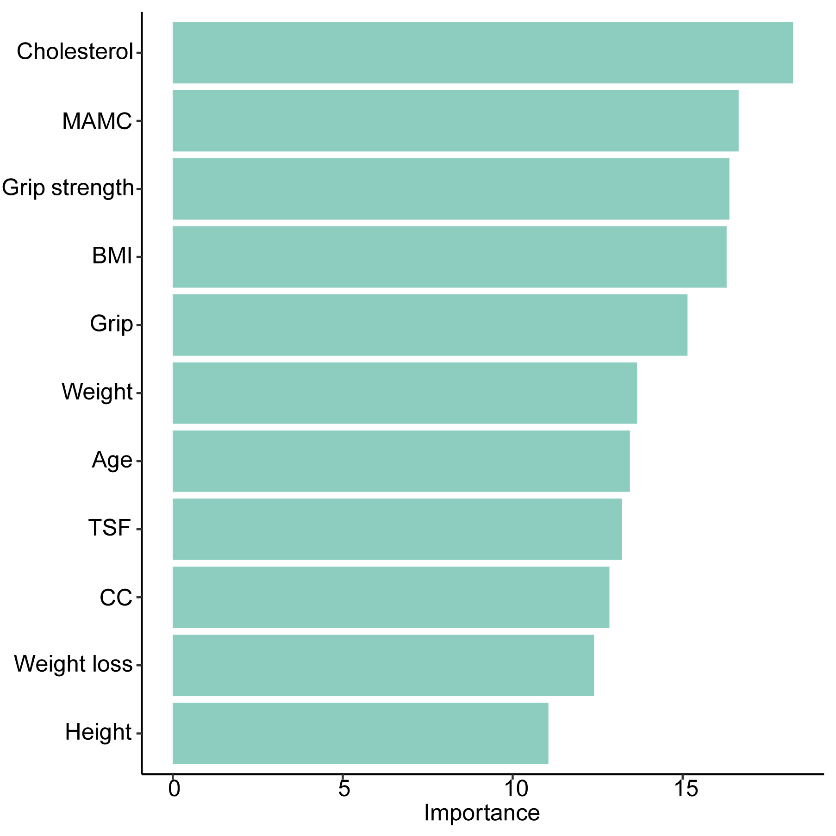


Figure S12. The association of CPNI and overall survival in patients with breast cancer.


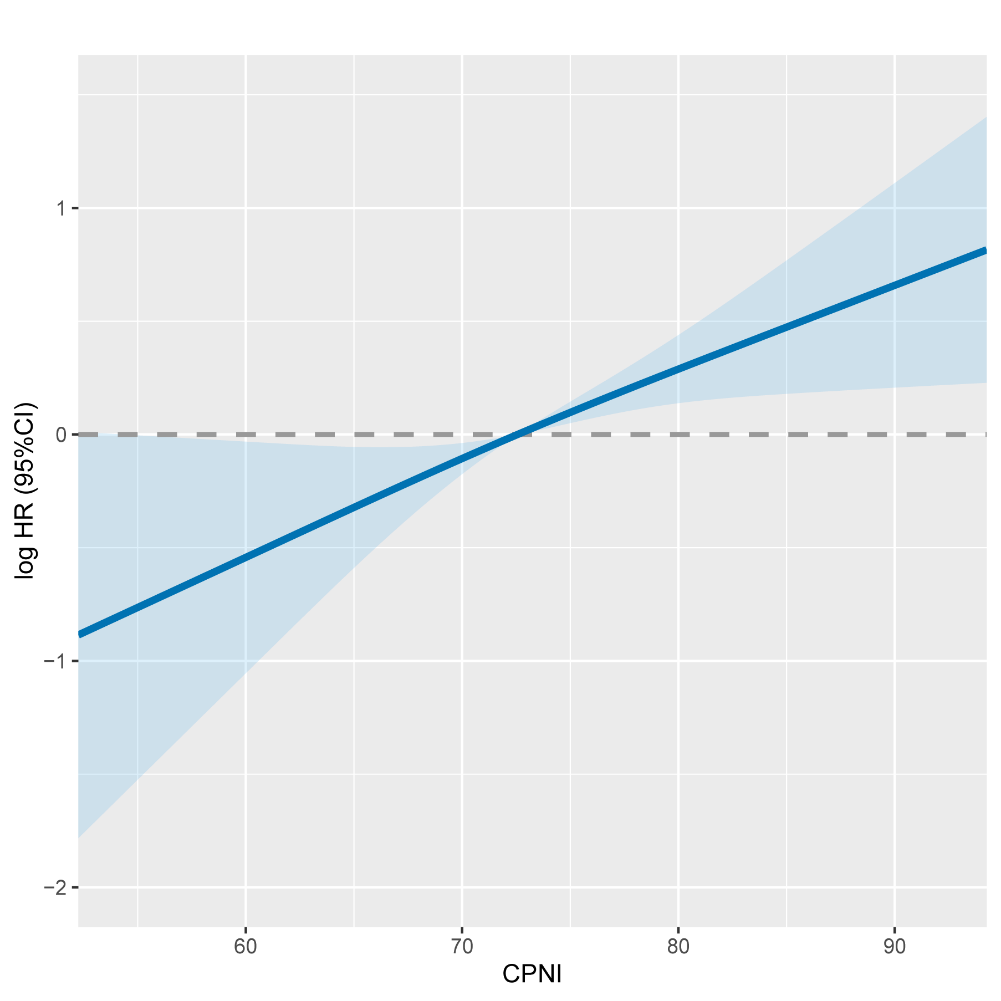


Figure S13. Cut-off values of CPNI in patients with breast cancer.


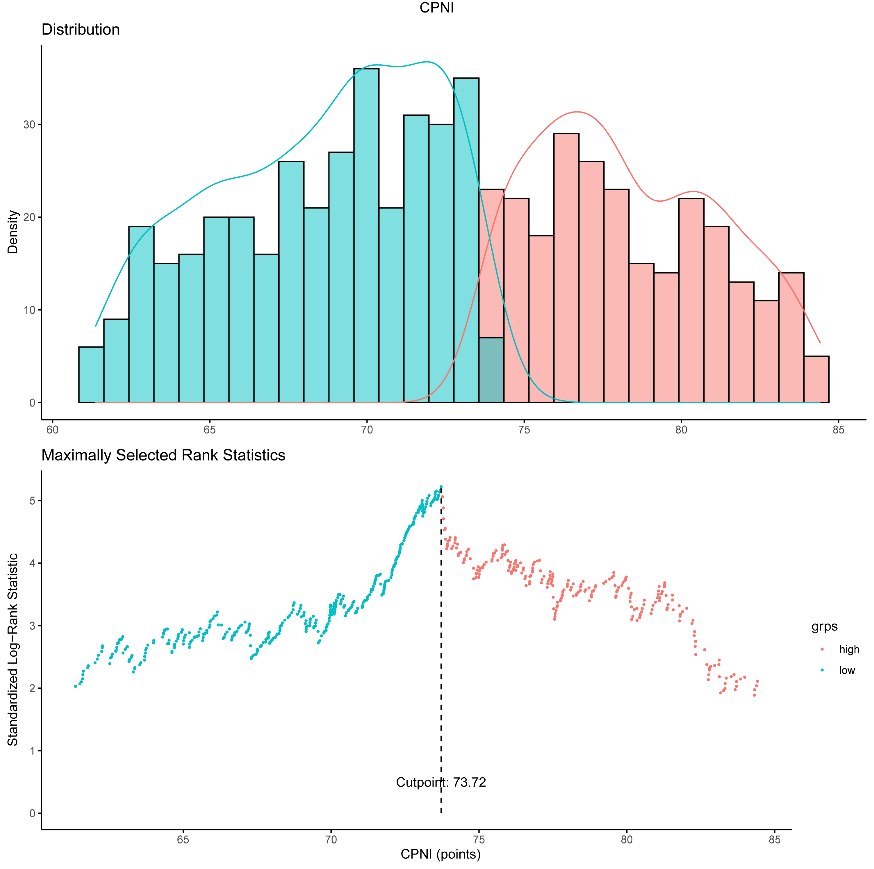


Figure S14. The time-dependent ROC of nutrition-relative indicators for diagnosing overall survival by excluding patients with short-term deaths (90-days).


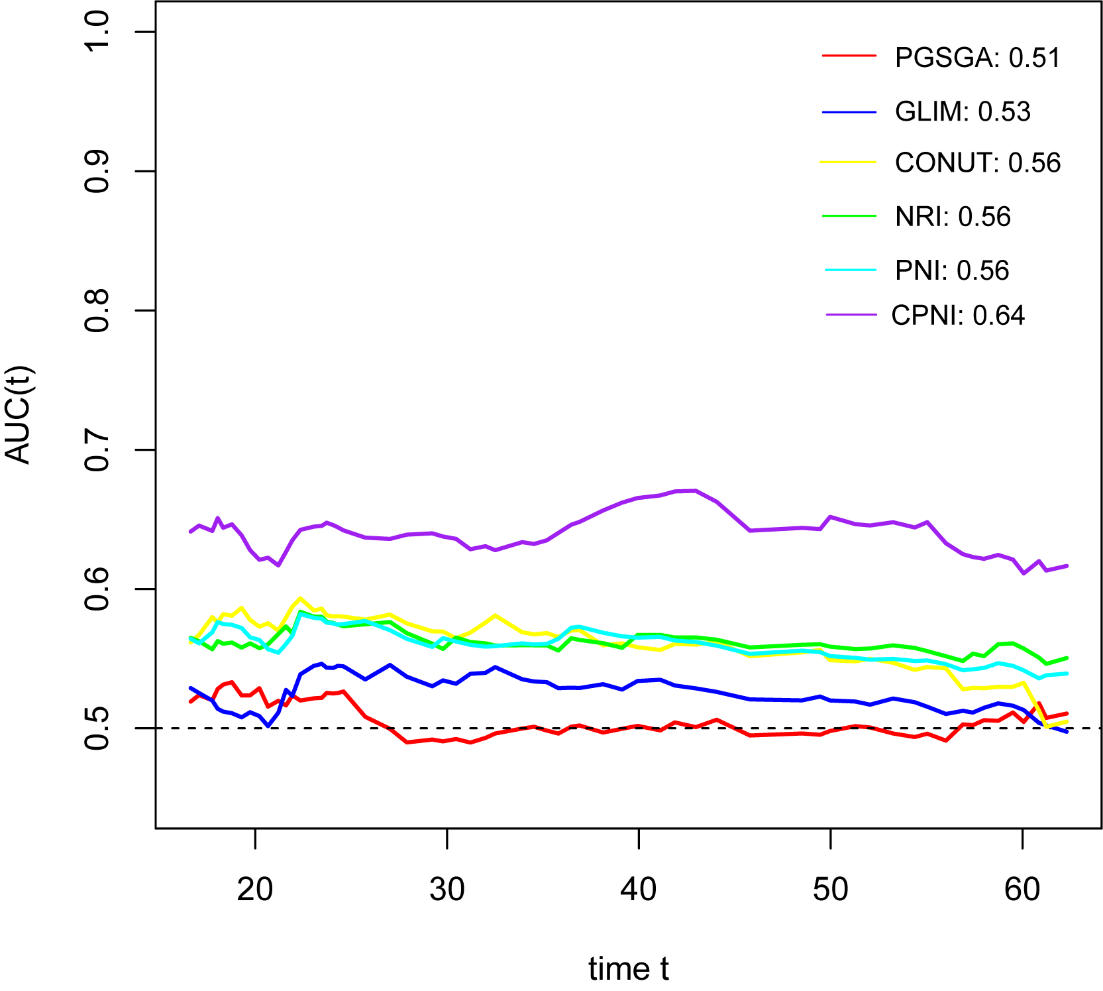


Figure S15. The time-dependent ROC of nutrition-relative indicators for diagnosing overall survival in patients with breast cancer.

Notes: A. train cohort. B. test cohort.


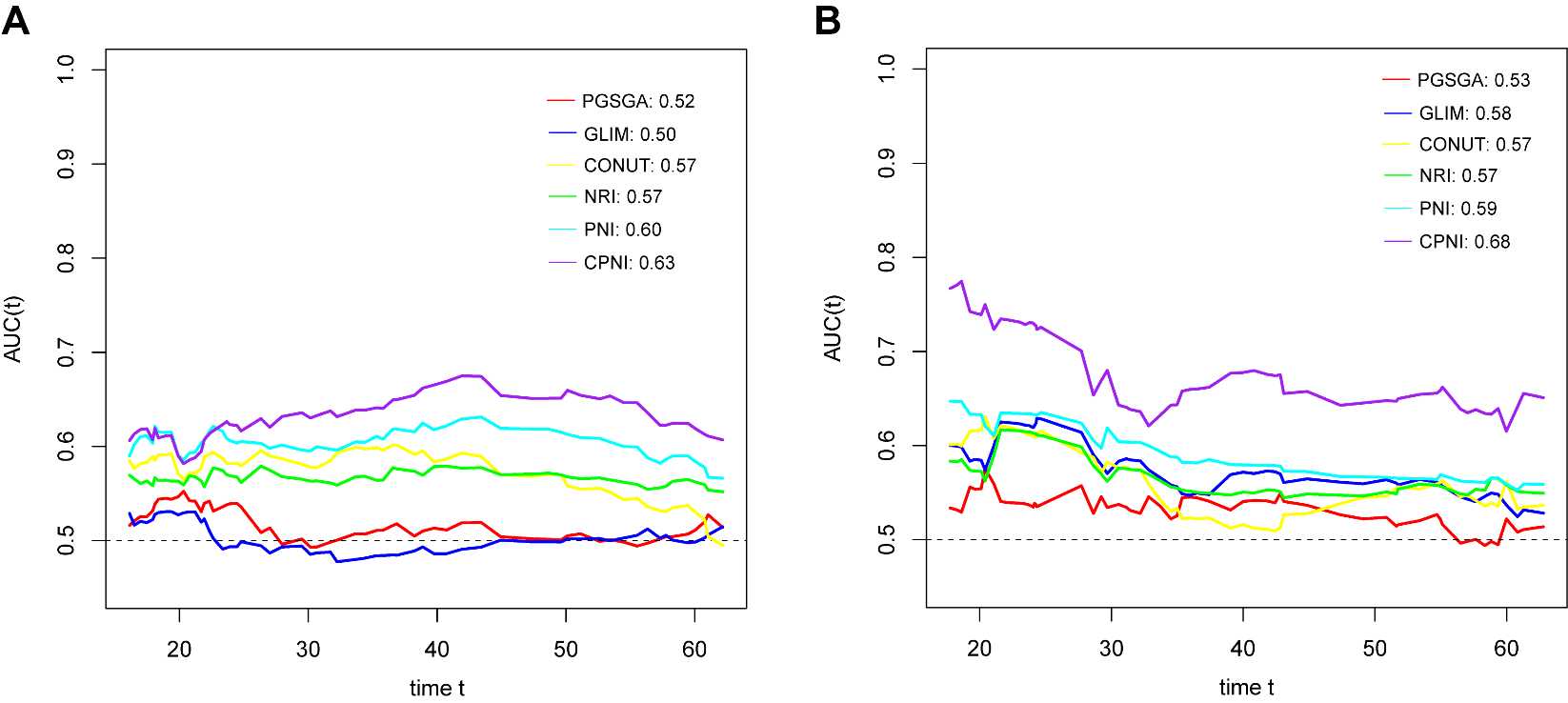


Figure S16. The sub-group analysis of the associations between nutrition indictors and mortality in patients with pre- and post-menopausal.


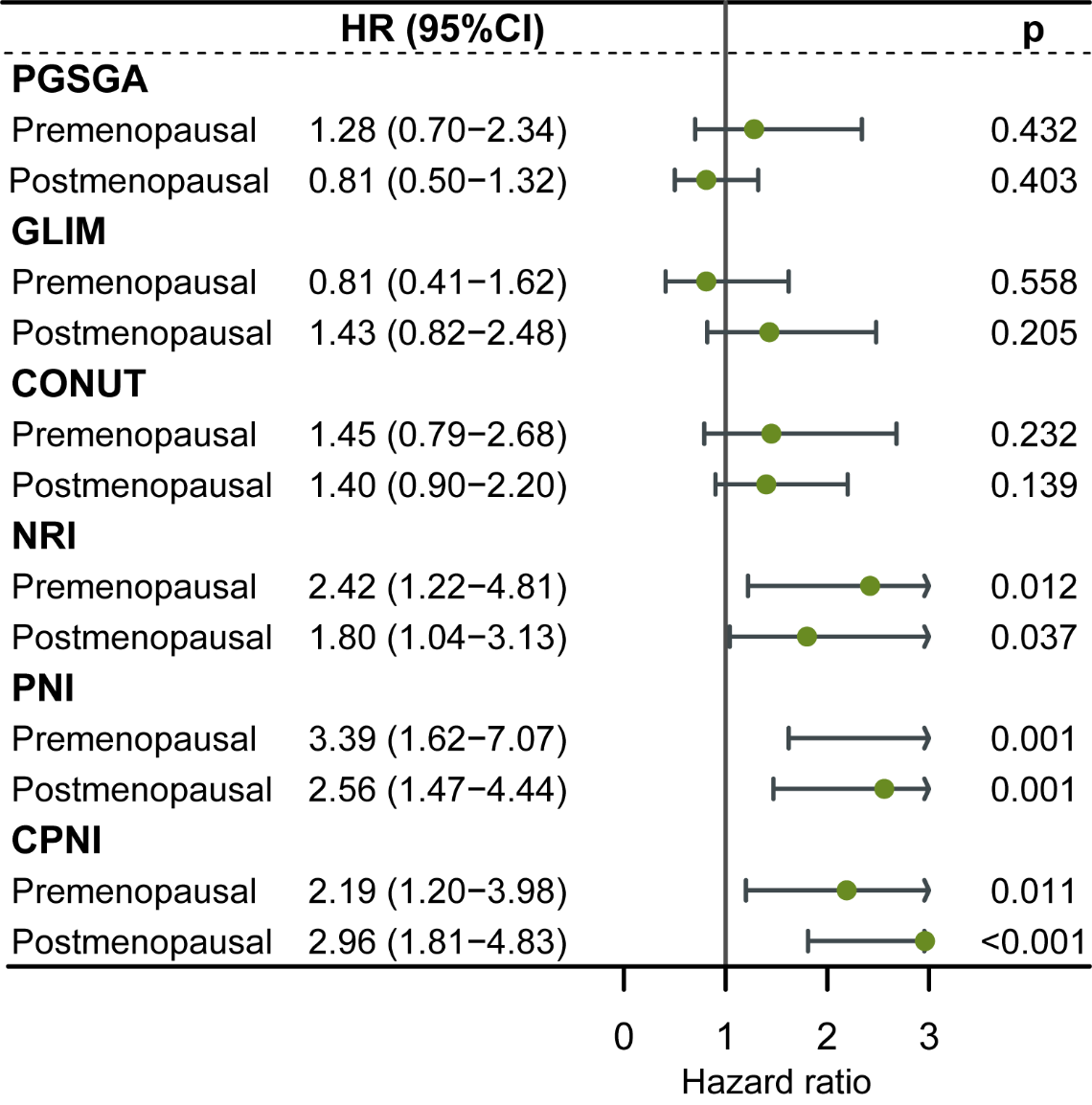


Figure S17. The sub-group analysis of the associations between nutrition indictors and mortality in non-overweight, overweight, and obese patients.


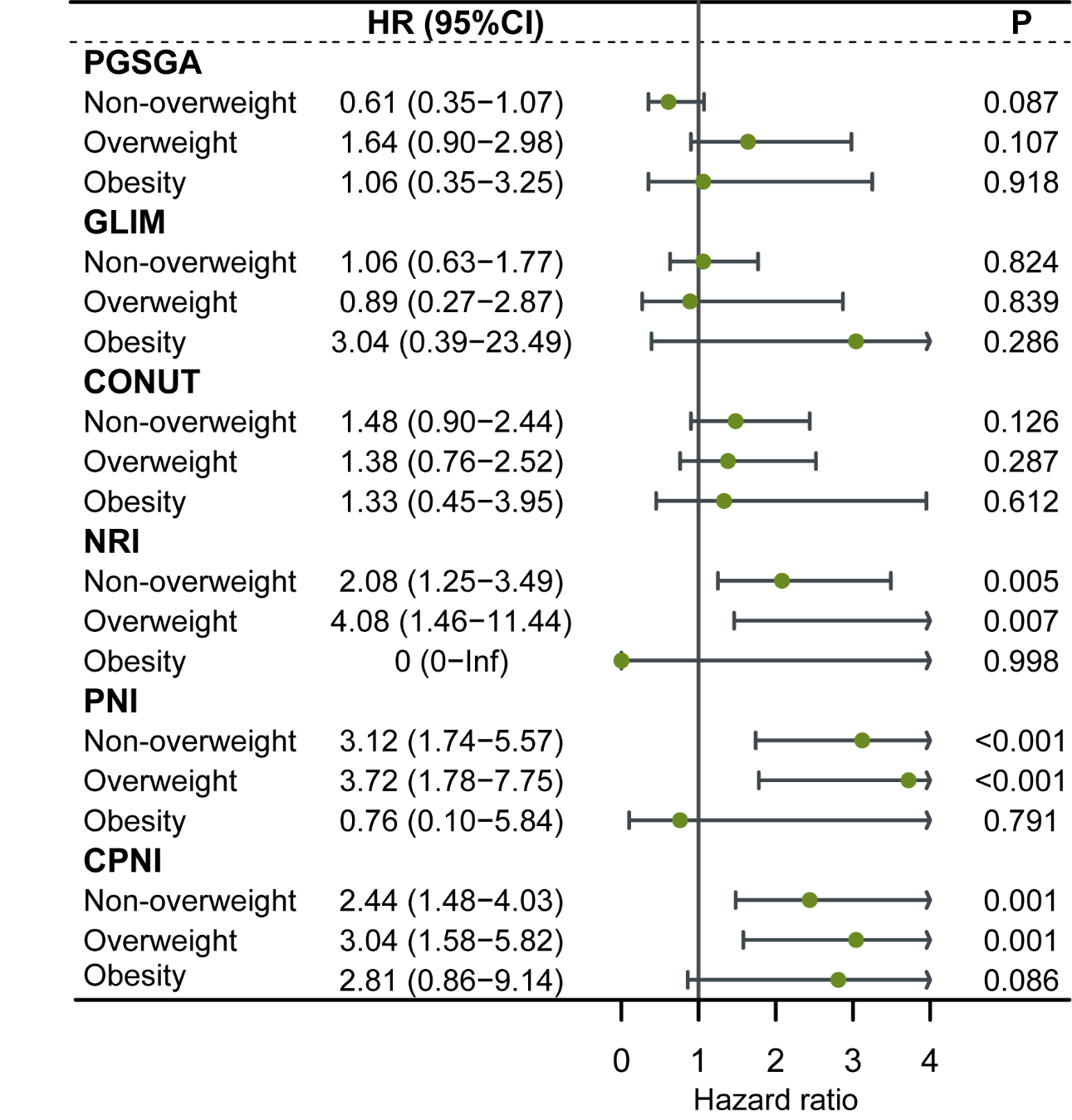
=

Table S1. Calculation methods for each nutrition indicator.

| **Indicators** | **Definition or calculation formula** |
| --- | --- |
| **PGSGA** | PGSGA nutritional score scale |
| **GLIM** | Meet at least one of the criteria |
| Weight loss (%) | >5% within the past 6 months, or >10% beyond 6 months |
| Low BMI (kg/m2) | <20kg/m2 if < 70 years, or <22 kg/m2 if ≥ 70 years |
| Reduced muscle mass | CC < p15, or weight standardized hand grip strength < p15, or MAMC < p15 |
| **CONUT** | Albumin score+ Lymphocyte score+ Cholesterol score |
| Albumin, g/L | Albumin score |
| 3.5-4.5 | 0 |
| 30-34.9 | 2 |
| 25-29.9 | 4 |
| <25 | 6 |
| Lymphocyte, *109/L | Lymphocyte score |
| ≥1.6 | 0 |
| 1.2-1.59 | 1 |
| 0.8-1.19 | 2 |
| <0.8 | 3 |
| Cholesterol, mmol/L | Cholesterol score |
| ≥180 | 0 |
| 140-180 | 1 |
| 100-139 | 2 |
| 100 | 3 |
| **NRI** | 1.519 × albumin (g/L) + 41.7 × current weight / IBW |
| **PNI** | Albumin (g/L) + 5 × lymphocyte count (×109) |
| **CPNI** | 4.8cholesterol-1.5 albumin - 7.7 lymphocyte + 126 |

Notes:

BMI=weight (kg) / height (m)2; IBW= [hight (m)]^2^×22; weight standardized hand grip strength = hand grip strength/weight.

The P15 of CC, weight standardized hand grip strength, and MAMC were 29 cm, 0.2144, and 17.06 cm in women.

BMI, Body Mass Index; CC, calf-circumference; MAMC, midarm muscle circumference; IBW, ideal body weight; PGSGA, the patient-generated subjective nutrition assessment; GLIM, the global leadership initiative on malnutrition; CONUT, the controlling nutritional status; NRI, the nutritional risk index, PNI, the prognostic nutritional index; CPNI, the cholesterol modified prognostic nutritional index.

Table S2. The AUC of nutrition indicators for all-cause mortality in patients with breast cancer.

|  | AUC | | |
| --- | --- | --- | --- |
| Indicators | 1-year | 3-year | 5-year |
| PGSGA | 0.555 | 0.515 | 0.485 |
| GLIM | 0.489 | 0.525 | 0.501 |
| CONUT | 0.601 | 0.575 | 0.518 |
| NRI | 0.601 | 0.569 | 0.554 |
| PNI | 0.637 | 0.585 | 0.546 |
| CPNI | 0.650 | 0.652 | 0.624 |
