# Supplementary material for: Cholesterol-modified prognostic nutritional index (CPNI) as an effective tool for assessing the nutrition status and predicting survival in patients with breast cancer
Source: BMC Med. 2023 Dec 21;21:512. doi: 10.1186/s12916-023-03225-7 (PMC10740286; doi:10.1186/s12916-023-03225-7)
Supplement: Supplementary file 2 — Additional file 2. CPNI Calcultator. [file 12916_2023_3225_MOESM2_ESM.html]

 


Calculator


# Calculator

Total Cholesterol
 mmol/L

Albumin
 g/L

Lymphocyte
 x109/L

**Score**

CPNI Score

Malnutrition

copyright © 2023 Hanping Shi. All Rights Reserved.
